# Inhibition of microRNA-155 Protects Retinal Function Through Attenuation of Inflammation in Retinal Degeneration

**DOI:** 10.1007/s12035-020-02158-z

**Published:** 2020-10-09

**Authors:** Riemke Aggio-Bruce, Joshua A. Chu-Tan, Yvette Wooff, Adrian V. Cioanca, Ulrike Schumann, Riccardo Natoli

**Affiliations:** 1 The John Curtin School of Medical Research, The Australian National University, Garran Road, Acton, Australian Capital Territory 2601 Australia; 2 The Australian National University Medical School, Mills Road, Australian Capital Territory, Acton, 2601 Australia

**Keywords:** Retina, miRNA, miR-155, Microglia, Macrophage, Inflammation, Photo-oxidative damage

## Abstract

Although extensively investigated in inflammatory conditions, the role of pro-inflammatory microRNAs (miRNAs), miR-155 and miR-146a, has not been well-studied in retinal degenerative diseases. We therefore aimed to explore the role and regulation of these miRNA in the degenerating retina, with a focus on miR-155. C57BL/6J mice were subjected to photo-oxidative damage for up to 5 days to induce focal retinal degeneration. MiR-155 expression was quantified by qRT-PCR in whole retina, serum, and small-medium extracellular vesicles (s-mEVs), and a PrimeFlow™ assay was used to identify localisation of miR-155 in retinal cells. Constitutive miR-155 knockout (KO) mice and miR-155 and miR-146a inhibitors were utilised to determine the role of these miRNA in the degenerating retina. Electroretinography was employed as a measure of retinal function, while histological quantification of TUNEL^+^ and IBA1^+^ positive cells was used to quantify photoreceptor cell death and infiltrating immune cells, respectively. Upregulation of miR-155 was detected in retinal tissue, serum and s-mEVs in response to photo-oxidative damage, localising to the nucleus of a subset of retinal ganglion cells and glial cells and in the cytoplasm of photoreceptors. Inhibition of miR-155 showed increased function from negative controls and a less pathological pattern of IBA1^+^ cell localisation and morphology at 5 days photo-oxidative damage. While neither dim-reared nor damaged miR-155 KO animals showed retinal histological difference from controls, following photo-oxidative damage, miR-155 KO mice showed increased a-wave relative to controls. We therefore consider miR-155 to be associated with the inflammatory response of the retina in response to photoreceptor-specific degeneration.

**Electronic supplementary material:**

The online version of this article (10.1007/s12035-020-02158-z) contains supplementary material, which is available to authorized users.

## Background

MicroRNAs (miRNAs) are short (~ 21–25 base pair), non-coding RNA regulators that are central to all biological processes [1]. In mammals, miRNAs are primarily transcribed into primary miRNA (pri-miRNA) within the nucleus where they are cleaved and exported into the cytoplasm to undergo further processing into the mature strand. Typically, regulation of mRNAs by a mature miRNA occurs through incorporation into Argonaute (AGO) proteins, most commonly AGO2, of the RNA-induced silencing complex (RISC) [2–4]. Although the mechanism of miRNA regulation and their targets under different biological and pathological conditions are still being elucidated, miRNAs have the capacity to simultaneously repress multiple mRNA targets within a specific process, such as oxidative stress and cellular inflammation [5, 6]. They are therefore ideal therapeutic targets for controlling dysregulated or overactive biological pathways [7, 8], such as occurs in retinal degenerations including age-related macular degeneration (AMD). Consequently, there is a growing focus on miRNAs as both diagnostics and therapeutics in chronic inflammatory conditions such as age-related macular degeneration (AMD) [7, 9–15].

miR-155 and miR-146a are two well-characterised miRNAs known for their roles in angiogenesis, oxidative stress and inflammation [16–23]. With approximately 45% sequence homology between them, miR-155 and miR-146a have been shown to act independently in and/or in tangent through the modulation of different processes involved in the innate immune response, including toll-like receptor (TLR), cytokine and complement signalling [21, 24–26]. As the Y402H mutation of the complement factor H (CFH) is the greatest genetic predictor of AMD development and severity [27], combinational suppression of miR-155 and miR-146a may be of particular interest due to their partially overlapping binging sites in the CFH mRNA 3′ untranslated region (UTR) [24, 28]. Unsurprisingly, both miR-155 and miR-146a have been shown to be upregulated in the blood and retinal tissue of AMD patients and animal models of retinal degeneration [29–32]. While the precise mechanism of miR-155 and miR-146a regulation and their influence on disease progression in the retina remains unclear, there is mounting evidence for a role in processes including mononuclear phagocyte (MP)–mediated inflammation [16, 18, 33, 34].

The activation and migration of MPs is a hallmark of many late-stage retinal degenerative diseases such as glaucoma [35], diabetic retinopathy [36], retinitis pigmentosa [37, 38] and AMD [39, 40]. Within the retina, the MP population is comprised of resident microglia, and in the degenerative state, further encompasses infiltrating blood-borne macrophages. MPs exhibit high plasticity, adopting a range of phenotypes along a functional and morphological spectrum. ‘Classical’ activation of MPs is amalgamated with the upregulation of pro-inflammatory cytokines, complement components and a dysregulation of modulatory molecules such as miRNAs [34, 39, 41]. The upregulation of miR-155, together with miR-146a, is consistently associated with this ‘classically’ activated MP profile; however, the immunomodulatory role of miR-146a in the MP response has not been clearly defined [41–46]. Regardless, due to their typical expression profile in degenerations, miR-155 and miR-146a have been suggested as therapeutic targets in numerous inflammation-associated conditions, such as systemic lupus erythematosus [47], psoriasis [48], rheumatoid arthritis and neurodegenerative conditions including, amyotrophic lateral sclerosis (ALS), Alzheimer’s disease and AMD [24, 26, 31, 49–51].

In this study we investigated the role of miR-155 and miR-146a in both the homeostatic and degenerating retina using a rodent photo-oxidative damage model, a light-induced model causing central photoreceptor cell death and increased inflammation [52]. We demonstrated that inhibition of miR-155 and miR-146a in combination results in loss of retinal function and increased inflammation. Subsequently, these miRNAs were investigated independently. While miR-146a did not alter the retinal response, inhibition of miR-155 resulted in functional protection, increased photoreceptor viability and reduced inflammation. We corroborated these findings through assessment of a miR-155 KO mouse, further demonstrating preservation of function and photoreceptor viability. MPs appeared to be the primary source of miR-155 in the degenerating retina degenerating retina but we also highlight the possibility of miR-155 being delivered to photoreceptors via small to medium extracellular vesicle (s-mEV). Consequently, we consider miR-155 to demonstrate a role in the inflammatory response of the degenerating retina, contributing to cell death and loss of function.

## Methods

### Animal Handling and Photo-oxidative Damage

All experiments were conducted in accordance with the ARVO Statement for Use of Animals in Ophthalmic and Vision Research. The study was approved by the Australian National University (ANU) Animal Experimentation Ethics Committee (Application ID: 2017/41). C57BL/6J (Stock No: 000664) and a miR-155 KO mouse line, C57BL/6-Mir155<tm1.1Ggard>/J (Stock No: 007745) from Jackson Laboratories (MA, USA) were utilised in this study. Mice were raised under dim (5 lx), 12-h cyclic light conditions, and were used as dim-reared controls in this study.

To induce photo-oxidative damage, adult (P60) C57BL/6J and miR-155 KO were exposed to 1, 3, or 5 days of 100 K lux natural LED with free access to food and water as previously described [52].

### Intravitreal Injections of miR-155 Inhibitors

miR-155-5p and miR-146a-5p inhibitors and a negative, scrambled control miRNA mimics (Thermo Fisher Scientific) were used for intravitreal injection. Prior to administration miRNAs were encapsulated into a cationic lipid-based transfection agent (Invivofectamine 3.0, Thermo Fisher Scientific) at a total concentration of 1 μg/μl, as described in a previous publication [53]. Intravitreal injections were performed according to previously described methodology [54]. At day 2 of a 5-day photo-oxidative damage paradigm, 1 μl of miRNA inhibitor or negative control miRNA-invivofectamine formulation was injected into both eyes of C57BL/6J mice. Animals were recovered for approximately 4 h prior to continuing photo-oxidative damage.

### Tissue Collection and Processing

Animals were euthanised by administration of CO_2_. Rodent eyes and retinas for histological processing and molecular analysis respectively were collected and processed as described in a previous publication [52]. Whole-mount retinal pigment epithelium (RPE) was collected from the whole eyes under a dissection microscope. The cornea and lens were removed, exposing the retina; the retina was then gently separated from the RPE/choroid using forceps and the RPE/choroid was collected for analysis.

Whole blood was collected from the left eye via retro-orbital bleeds. Serum was separated by clotting samples at RT for 20 min followed by centrifugation at 1000×*g* for 10 min at 4 °C.

### Flow Cytometry

Rodent retinas of adult C57BL/6J mice were digested using the Neural Tissue Dissociation Kit - Postnatal Neurons (Miltenyi Biotec, NRW, Germany, 130-094-802) according to the manufacturer’s instructions for manual dissociation of retinas. Dissociated cells were re-suspended in a 1% Bovine Serum Albumin (BSA, Thermo Fisher Scientific, MS, USA, AM2616) in Phosphate-Buffered Saline (PBS, Thermo Fisher Scientific, 10010023) solution and passed through a 4% BSA cushion by centrifugation at 100×*g* for 10 min at 4 °C.

Anti-mouse, fluorochrome-conjugated monoclonal antibodies, FITC-Vimentin (1:5, Santa Cruz, TX, US, sc-32,322 FITC), PE-Rhodopsin (1:2500, Santa Cruz, sc-57,432 PE), PE/CY7-CD11b (1:500, Biolegend, CA, USA, 101215) and BV605-CD90.2 (1:250, Thy1.2, Biolegend, 140317) were stained at room temperature for 30 min to identify retinal cell populations, as per the PrimeFlow™ assay protocol. DAPI (stored at 1 mg/ml, used 1:10000, Sigma-Aldrich, D9542-1MG) and 7-AAD (1:1000, Biolegend, 420403) were added immediately prior to flow cytometry as nuclear stains for identification of viable cells.

To detect miR-155 in retinal cells against surface markers, PrimeFlow™ assay (Thermo Fisher Scientific, 88-18005-210) with microRNA pre-treatment (Thermo Fisher Scientific, 88-18006) protocol was performed according the manufacturer’s guidelines and using a type 1, Alexa Fluor 647-labelled miR-155-5p (Thermo Fisher Scientific, VM1-10229-PF), and miR-124-3p (Thermo Fisher Scientific, VMI-10271-PF) as a biological control.

Data was acquired by both standard flow cytometry (BD LSRFortessa™) using the BD FACSDiva Software (BD Biosciences, NJ, USA) and imaging flow cytometry (Amnis® ImagStream®) using the Amnis INSPIRE® Software (Luminex corporation, TX, USA). Data was analysed using the FlowJo (BD Biosciences) and Amnis IDEAS® (Luminex corporation) software respectively. Cytoplasmic spot counting was performed by creating a cytoplasmic mask of the intracellular, DAPI-negative area of rhodopsin positive cells. This mask was combined with the spot counting feature to obtain counts of miR-155 expression within the cytoplasm.

### Small–Medium Extracellular Vesicle Isolation

S-mEVs were isolated from papain-digested retinas by sequential ultracentrifugation following the MISEV2018 guidelines for studies of extracellular vesicles [55] and adapted for retina [56]. Briefly, retinas were mechanically and enzymatically dissociated in a digestion solution of 2.5 mg/ml papain (Worthington Biochemicals), 200 U DNAse I (Roche Applied Science, Penzberg, Germany) 5 μg/ml catalase (Sigma-Aldrich), 10 μg/ml gentamycin (Sigma-Aldrich) and 5 μg/ml superoxide dismutase (Worthington Biochemicals) in Hank’s Balanced Salt Solution (HBSS; Gibco; Thermo Fisher Scientific). Enzymatic digestion was performed at 37 °C for 8 min, followed by 20 min at 8 °C, to allow for s-mEV release. Following digestion, tissue suspensions were neutralised by dilution in 11.5 ml of HBSS (Thermo Fisher Scientific) and centrifuged at 1000×*g* for 10 min at 4 °C to allow for the removal of cells and debris. The supernatant was transferred to 14 × 89 mm Beckman Ultra-Clear ultracentrifuge tubes and spun down at 10,000×*g* for 30 min in a Beckman Coulter Optima XE-100 fitted with a SW41Ti Rotor. This step allowed for the precipitation of large EVs (lEV) and remaining cell debris.

The s-mEV-containing supernatant was transferred to new ultracentrifuge tubes and centrifuged for a further 1.5 h at 150,000×*g*. The supernatant was carefully decanted, and the s-mEV pellet was resuspended via tituration for 1 min. For s-mEV RNA analysis, the s-mEV pellet was resuspended in 100 μl of RNase A at 10 μg/ml (EN0531, Thermo Fisher Scientific) and incubated for 30 min at 37 °C to remove external RNA contamination. Following RNase treatment, s-mEV RNA was extracted using a mirVana miRNA isolation kit (Thermo Fisher Scientific) according to the manufacturer’s instructions.

### Electroretinography

Retinal function was assessed using full-field scotopic electroretinography (ERG) as described previously [52]. Briefly, mice were dark-adapted overnight, anesthetised by intraperitoneal injection of ketamine (100 mg/kg) and xylazil (10 mg/kg), and pupils dilated with a single drop of 1% atropine sulphate. A single- or twin-flash paradigm was used to elicit mixed (rod and cone) responses, respectively. Flash stimuli for mixed responses were provided by an LED-based system (FS-250A Enhanced Ganzfeld; Photometric Solutions International, Huntingdale VIC, Australia), across a stimulus intensity range of 6.3 log cd s m^−2^ (range, − 4.4 to 1.9 log cd s m^−2^).

### Haematoxylin and Eosin Staining

Thawed retinal cryosections were moved through an ethanol gradient of 95% for 2 min and 70% for 3 min. Slides were rinsed in milli-Q water and covered with Harris Haematoxylin solution (Sigma-Aldrich) for 2 min. Sections were rinsed in Scott’s tap water (2 g/l sodium bicarbonate and 20 g/l magnesium sulphate in milli-Q water), and Eosin Y counterstain (Sigma-Aldrich) was applied for 2.5 min. Slides were dehydrated through an ethanol gradient starting at 70% for 3 min, 95% for 1 min and 100% for 1 min. Slides were coverslipped using DPX (Sigma-Aldrich).

### TUNEL

Retinal cryosections were labelled for cell death using a terminal deoxynucleotidyl transferase dUTP nick end labelling (TUNEL) kit (Roche Applied Science), as described previously [57]. For quantification of photoreceptor cell death, TUNEL^+^ cells in the outer nuclear layer (ONL) were counted along the full length of the retinal section, cut in the parasagittal plane. Cumulative cell death was determined by ONL thickness measurements at 600-μm increments from the optic nerve head along the full length of the retinal cryosection. For each animal, counts/measures were performed in technical duplicate, averaging for each experimental group.

### Immunolabelling

Immunohistochemical analysis of retinal cryosections was performed as described previously [58]. Microglia/macrophages were immunolabelled with primary mouse anti-rabbit ionised calcium-binding adaptor molecule 1 (IBA1; 1:500, cat. 019-19741, Wako, Osaka, Japan) and secondary goat anti-rabbit Alexa 488 (1:500, cat. A11034, Thermo Fisher Scientific).

Quantification of IBA1^+^ cells (marker of retinal MPs) was performed across the superior and inferior retina of cryosections. The number of IBA1^+^ cells in the outer retina was quantified as the total counts in the ONL and subretinal space. Nuclear layers were visualised by staining cryosections with the DNA-specific dye, Hoechst 33342 solution (20 mM, 1:10000, Thermo Fisher Scientific). The ONL thickness was measured at increments of 600 μm across the length of the retinal cryosection, including the optic nerve head.

RPE integrity was determined by staining RPE/choroid whole mounts with an Alexa Fluor™ 594 conjugated phalloidin probe (1:100, Thermo Fisher Scientific, A12381) for 2 h at room temperature (RT).

### Confocal Imaging

Fluorescence was visualised and captured by using a ZEISS LSM800 with Airyscan Super-resolution confocal microscope (ZEISS Microscopy, BW, Germany). Excitation wavelengths of 488 nm (green), 561 nm (red) and 358 nm (Hoechst) were used for image acquisition. Images were obtained using uniform gain settings and processed using the ZEISS Zen (blue edition) software and ImageJ (NIH, MA, US). H&E staining was captured using the brightfield setting of a laser-scanning A1 confocal microscope (Nikon Instruments, Inc., Tokyo, Japan). High-magnification images of retinal histology were taken as representative images from the superior retina at 500 μm from the optic nerve head as reported previously [52].

### Measures of miRNA and mRNA Expression

Extraction and purification of mRNA was performed on isolated retinas using a combination of TRIzol reagent (Thermo Fisher Scientific, Inc., MA, USA) and the RNAqueous Total RNA isolation kit (Thermo Fisher Scientific), according to previously published methodology [57]. Extraction and purification of miRNA was performed using the miRVana miRNA isolation kit (Thermo Fisher Scientific) following the manufacturer’s instructions.

From a mRNA template, cDNA synthesis was performed using the Tetro cDNA Synthesis Kit (Bioline Reagents, London, UK) as described previously [58]. From a miRNA template, cDNA was synthesised using the TaqMan MicroRNA RT kit (Thermo Fisher Scientific) with the appropriate RT Single TaqMan® Assay (Thermo Fisher Scientific; Table 1). Quantitative real-time PCR (qRT-PCR) was performed using TaqMan hydrolysis probes (Table 1). Reactions were run on a QuantStudio Flex 12 K instrument (Thermo Fisher Scientific) and with each sample in technical duplicate. The average cycle threshold (Ct) values were used to determine expression. Percent expression change was calculated using the comparative Ct (ΔΔCt) method, where targets were normalised to the expression of a reference of *Gapdh* and *Sdha* for mRNA and small nuclear RNA U6 and small nucleolar RNA 234 for miRNA as per previous analyses [59, 60]. The average Ct for each probe in different tissues is included in Supplementary Table 1).

**Table 1:** TaqMan® Probes

Gene symbol	Name	Catalogue number
*Antxr2*	Anthrax toxin receptor 2	Mm01203285_m1
*Bdnf*	Brain-derived neurotrophic factor	Mm04230607_s1
*Gapdh*	Glyceraldehyde-3-phosphate dehydrogenase	Mm99999915_g1
*Il-6st*	Interleukin 6 signal transducer	Mm00439665_m1
*Il-10*	Interleukin 10	Mm01288386_m1
*miR-155*	mmu-miR-155-5p	002571 (assay ID)
*miR-146a*	hsa-miR-146a-5p	000468 (assay ID)
*Sdha*	Succinate dehydrogenase complex, subunit A	Mm01352366_m1
*snoRNA234*	Small nucleolar RNA 234	001973 (assay ID)
*Socs1*	Suppressor of cytokine signalling 1	Mm00782550_s1
*Tnf-α*	Tumour necrosis factor alpha	Mm00443259_m1
*U6 snRNA*	U6 small nuclear RNA	001234 (assay ID)

### Identification of AGO Bound Targets

Potential miR-155-5p mRNA targets were elucidated based on previously published Argonaute immunoprecipitation (Ago IP) data [61]. Potential targets were selected based on demonstration of a significant negative log-fold change in response to photo-oxidative damage determined using a linear model fit and Bayes adjustment of *p* values. The mRNA database is available from BioProject (accession ID: PRJNA606092).

### Cell Culture

Murine photoreceptor–derived 661W cells (kindly gifted by Dr. Muayyad R. Al-Ubaidi, Dept. of Cell Biology, University of Oklahoma Health Sciences Centre, Oklahoma City, OK, USA) [62], murine brain–derived microglia C8B4 (American Tissue Culture Collection (ATCC), Virginia, USA) [63], immortalised human Müller-like MIO-M1 (Moorfield’s Institute of Ophthalmology, London, UK) [64] and immortalised human RPE-like aRPE19 (ATCC) [65] were validated for species authenticity (CellBank, Sydney, Australia), and were all used for experimental purposes within 10 passages. Cells were cultured in Dulbecco’s Modified Eagle’s Medium (DMEM, Thermo Fisher Scientific) containing 10% fetal bovine serum (FBS), l-glutamine (3 mM), sodium pyruvate (1 mM) and glucose (25 mM) and grown in flasks in a humidified incubator set to 37 °C with 5% CO_2_.

### Cell Culture Stimulation

A total of 661W photoreceptor-like cells were seeded in 24-well plates (Nunc, Thermo Fisher Scientific, MA, USA) at 2.5 × 10^5^ cells/well, 36 h prior to photo-oxidative damage exposure. Twenty-four hours following seeding, culture medium was replaced with a reduced (1%) serum medium of 1% FBS. A total of 661W cells were exposed to 15,000 lx light (2.2 mW/cm^2^; irradiance measured with PM100D optical power metre, THORLABS, NJ, USA) from two white fluorescent lamps (2 × 10 W T4 tri-phosphor 6500 K daylight fluorescent tubes; Crompton, NSW, Australia), for 5 h with 5% CO_2_ at 37 °C to induce photo-oxidative damage. For dim control cells, one plate was completely wrapped in aluminium foil to avoid light exposure. For air/gas exchange, six small incisions were cut on the aluminium foil. Cells were then washed with 1× PBS and extracted using the mirVana™ miRNA extraction kit according to the manufacturers’ protocol.

C8-B4 cells and MIO-M1 cells were seeded in 24-well plates at a density of 15 × 10^4^ and 2 × 10^4^ respectively. Twenty-four hours following seeding, the culture medium was replaced with a reduced (1%) serum medium. Thirty-six hours following seeding, cells were challenged with a cytokine cocktail of 10 ng/ml interleukin 1β (IL-1β; R&D Systems, MN, USA), 10 ng/ml tumour necrosis factor α (TNF-α; R&D Systems) and 100 U/ml interferon γ (IFN-γ; R&D Systems), as reported previously [19], for 24 h to elicit an inflammatory response. Following cytokine stimulation, adherent cells were washed with 1× PBS and extracted using the mirVana™ miRNA extraction kit according to the manufacturer’s protocol.

### 3′UTR CFH Luciferase Assay

A luciferase assay was performed on HeLa cells using a commercially available vector containing the luciferase gene fused to the human *CFH* 3′UTR (*CFH* LightSwitch 3′UTR Reporter GoClone; Switchgear Genomics, CA, USA) and a pLightSwitch-3′UTR Luciferase assay system (Switchgear Genomics), following the manufacturer’s protocol. Control cells were transfected with a scrambled-sequence miRNA.

HeLa cells were seeded in a 96-well plate (5000 cells per well), cultured for 24 h, following which the pLightSwitch Luciferase assay system was performed according to the manufacturer’s protocol. Luciferase activity was measured using an Infinite 200 plate reader (TECAN, Grödig, Austria).

### Statistical Analysis

For experiments compared between two groups, data was assessed for normal distribution using a Shapiro-Wilk test, and statistical significance was determined by an unpaired Student’s *t* test. For group comparisons, a 1-way ANOVA with Tukey’s post hoc test was used to determine significance. For ERG and ONL thickness analysis, a 2-way ANOVA with Sidak’s post hoc test for multiple comparisons was applied. All statistical analysis was performed using GraphPad Prism V7 (GraphPad Software, La Jolla, CA, USA). Statistical significance was determined by *p* < 0.05.

## Results

### Targeted Inhibition of miR-155 Alleviated Degeneration in the Photo-oxidative Damage Retina

**Fig. 1:**
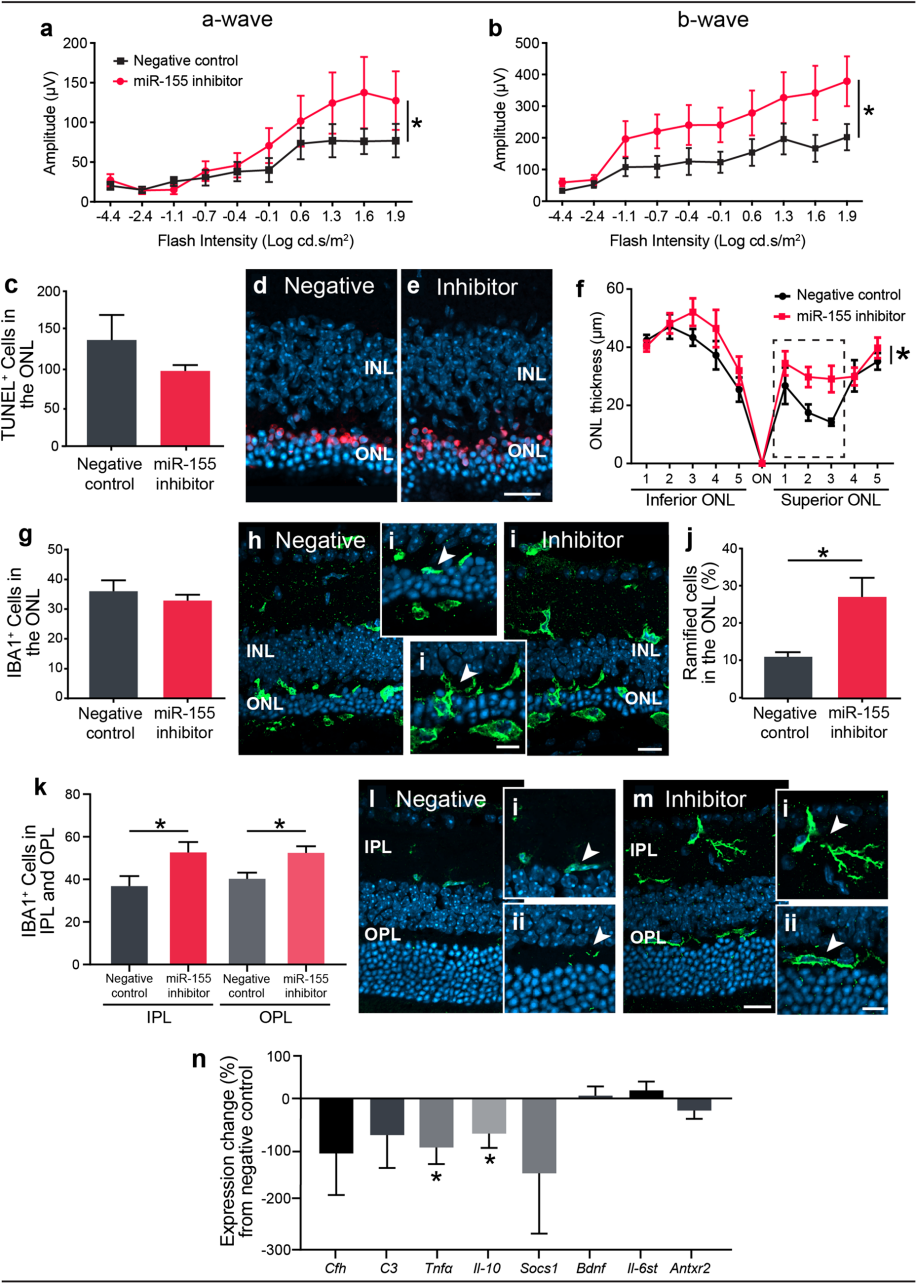
Inhibition of miR-155 was protective against photo-oxidative damage (PD)–induced degeneration. **a**–**b** miR-155 inhibitor–injected retinas demonstrated a significantly greater function, as measured by ERG, with a higher (**a**) a-wave and (**b**) b-wave amplitude compared with the negative control (*p* < 0.05). **c–e** Quantification of photoreceptor cell death by TUNEL labelling demonstrated no significant difference in TUNEL^+^ cell counts between groups. **d**–**e** Representative images displaying TUNEL immunolabelling of retinal cryosections. **f** ONL thickness measured across the length of the retina showed a significantly thicker ONL in the miR-155 inhibitor mice (*p* < 0.05). The box indicates the region of focal cell death. **g–l** IBA1^+^ cells were quantified in miR-155 inhibitor– and negative control–injected retinas which showed (**g**) no significant difference in total number of IBA1^+^ cells between groups. **h–i** Representative images of IBA1^+^ cells infiltrating the ONL. **j** Within the outer retina, miR-155 inhibitor–injected retinas showed a significantly greater percentage of ramified IBA1^+^ cells (*p* < 0.05). **k**–**m** Significantly, more IBA1^+^ cells were found within both the IPL and OPL of the retina in miR-155 inhibitor–injected mice compared with that in negative controls (*p* < 0.05). **l**–**m** Representative images show IBA1^+^ cells in the IPL and OPL of the peripheral retina. **n** Gene expression of *Cfh*, *C3*, *Il-10*, *Tnfα* and *Socs1*, and predicted targets of miR-155 (*Bdnf*, *Il6st* and *Antxr2*) were analysed by qRT-PCR. No significant difference was seen in the gene expression of *Cfh*, *C3 Socs1*, *Bdnf*, *Il6st* or *Antxr2* (*p* > 0.05); however, the expression of *Il-10* and *Tnfα* was significantly decreased in miR-155 inhibitor–injected retinas (*p* < 0.05). Statistical significance was determined by Student’s *t* test and two-way ANOVA with post hoc multiple comparison (*n* = 10 animals per group, *represents *p* < 0.05). ONL, outer nuclear layer; INL, inner nuclear layer. For all images, scale bars represent 20 μm (Hi, Ii: scale bar represents 10 μm)

Previous findings have identified immune cell infiltration into the ONL to increase at 3 days photo-oxidative damage, reaching peak infiltration at 5 days [52]. Therefore, to target the MP inflammatory response, a miR-155 inhibitor or a negative scrambled miRNA control was injected intravitreally into mouse retinas at day 2 of a 5-day photo-oxidative damage experimental paradigm. Retinas treated with miR-155 inhibitor had improved function resulting from negative miRNA inhibitor–injected controls, as indicated by a significantly lower amplitude across all flash intensities for both the a-wave and b-wave responses (Fig. 1a–b). The protective effect of miR-155 inhibitors was further investigated, which typically show a progressive increase in TUNEL and a decrease in ONL thickness over the period of retinal degeneration [52]. There was no significant reduction in the number of TUNEL^+^ cells in the ONL (Fig. 1c–e). However, there was significantly less ONL thinning in miR-155 inhibitor–injected retinas compared with that in scrambled miRNA inhibitor controls (Fig. 1f), indicating a cumulative protection against photoreceptor cell death.

IBA1^+^ immunostaining displayed no decrease in the total number of MPs recruited to the outer retina between mice treated with miR-155 inhibitors and scrambled miRNA inhibitor controls (Fig. 1g–i). However, there were proportionately more IBA1^+^ cells in the outer retina of miR-155 inhibited retinas (average of 28% compared with 10%) that exhibited a ramified, homeostatic morphology (Fig. 1j). There was also a greater number of IBA1^+^ cells present in both the inner plexiform layer (IPL) and outer plexiform layer (OPL) in the miR-155 inhibitor–treated retinas (Fig. 1k).

As *Cfh* is a reported target of miR-155, complement targets were investigated by qRT-PCR demonstrating no significant difference in the expression of *Cfh* or the downstream molecule *C3* (Fig. 1n). Therefore, we used AGO2 immunoprecipitation to identify AGO2-bound potential alternative targets of miR-155 (Supplementary Table 2). Three predicted targets, *Bdnf*, *Antxr2* and *Il-6st*, were chosen for further investigation based on a significant expression change following photo-oxidative damage in addition to potential roles in neuroprotection [66], angiogenesis [67] and cytokine signalling [68] respectively. These targets were explored in conjunction with the pro-inflammatory cytokine *Tnfa* and the immunomodulatory *Il-10* and *Socs1*. In the miR-155-inhibited retina, significantly lower levels of *Tnfa* and *Il-10* were recorded by qRT-PCR, when compared with the negative inhibited control (Fig. 1n). However, there was no significant increase in the levels of *Socs1* or the AGO2-bound potential targets *Bdnf*, *Il-6st* or *Antxr2* (Fig. 1n).

As non-traditional immune cells, RPEs have been shown to express miR-155 under stress [19]. Here, the relationship between miR-155 and the degenerating RPE was further explored by measuring RPE integrity in the photo-oxidative damage model following miR-155 inhibition. There was no significant decrease in the lesion size or the number of adherent subretinal MPs in the RPE following inhibition of miR-155 compared with negative inhibitor controls (Supplementary Fig. 1). Furthermore, there was no change in IBA1^+^ cells or gene expression for the measured targets. This suggests that although inhibition of miR-155 demonstrates functional protection in the retina, no protective effect appears to extend to the RPE.

### MiR-155 KO Retinas Do Not Exhibit Functional or Obvious Histological Differences

**Fig. 2:**
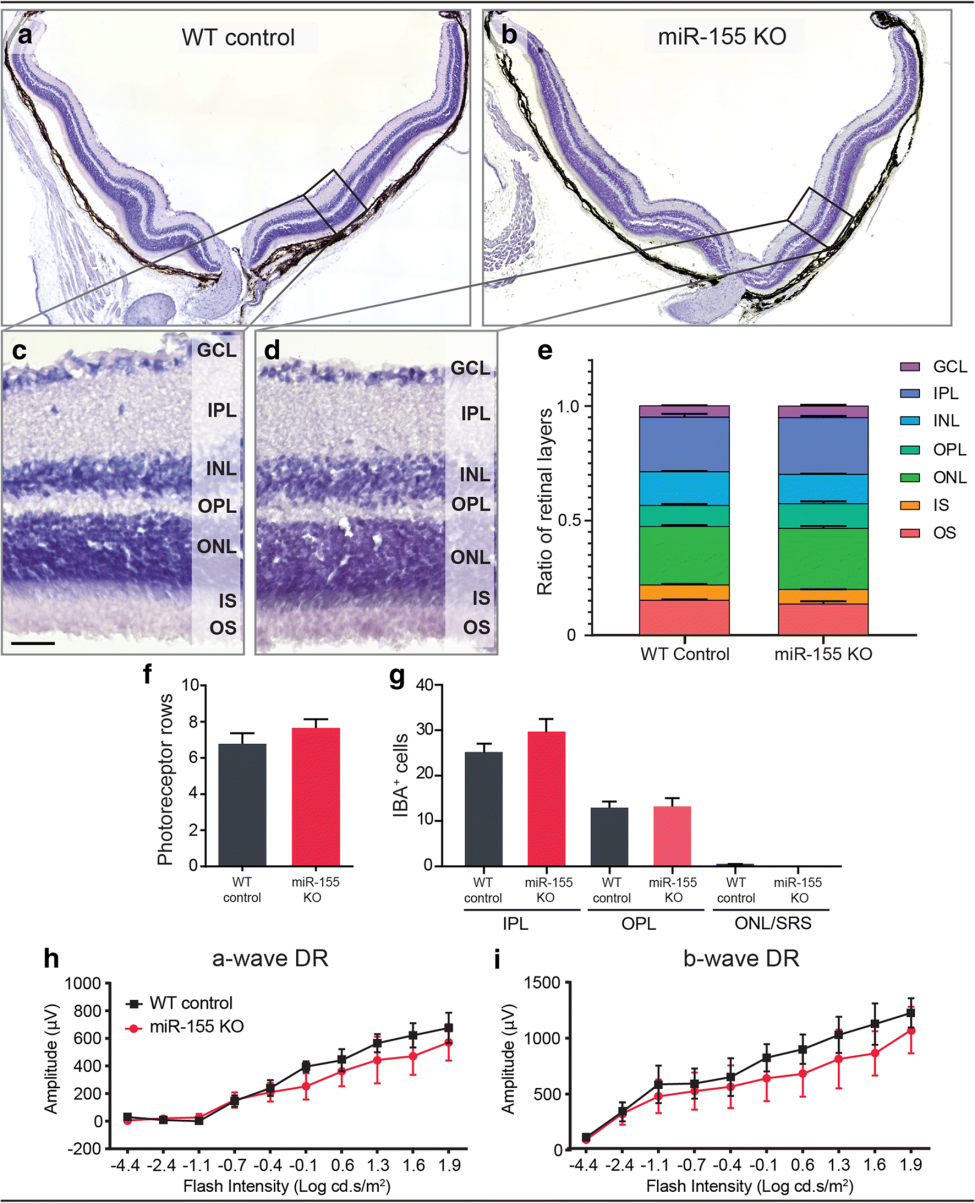
DR miR-155 constitutive knockout (KO) retinas demonstrated no difference compared with that of WT controls. **a**–**d** Representative images of (**a**–**b**) H&E-stained whole retinas (**c**–**d**) and a region of superior retina approximately 600 μm from the optic nerve. **e** Thickness of retinal layers represented as a ratio of each layer to total retinal thickness indicated no significant difference in thickness of retinal layers between WT and miR-155 KO DR mice. Furthermore, no difference was observed in (**f**) photoreceptor row counts in the superior retina of control and miR-155 KO mice (*p* > 0.05). **g** The number of IBA1^+^ cells in the IPL, OPL and the ONL/SRS was quantified in control and miR-155 KO retinas and showed no significant difference between groups (*p* > 0.05). **h**–**i** ERG recordings of DR miR-155 KO and control mice recorded over a range of flash intensities showed no significant difference between groups for either the (**h**) a-wave or the (**i**) b-wave response (*p* > 0.05). Statistical significance was determined by Student’s *t* test and two-way ANOVA with post hoc multiple comparison (*n* = 6 animals per group, *represents *p* < 0.05). GCL, ganglion cell layer; IPL, inner plexiform layer; INL, inner nuclear layer; OPL, outer plexiform layer; ONL, outer nuclear layer; IS, photoreceptor inner segments; OS, photoreceptor outer segments; SRS, sub-retinal space. For all images, scale bars represent 20 μm

A miR-155 KO mouse was used to further demonstrate the role of miR-155 in the retina, and retinal morphology including the thickness of retinal layers was assessed compared with aged-matched C57BL/6J WT controls (Fig. 2a–d). Results showed no significant difference in the thickness of retinal layers between WT and miR-155 KO mice (Fig. 2e). Additionally, no difference in the number of rows of photoreceptor nuclei in the ONL was observed (Fig. 2f). As miR-155 is known to influence the activation state of MPs [44, 69], the number of IBA1^+^ cells in the plexiform layers, ONL and subretinal space (SRS) was quantified. However, no significant difference was observed in the number of IBA1^+^ cells in these layers (Fig. 2g). Finally, the effect of miR-155 deficiency on retinal function was measured by ERG. Functionally, there was no difference in the a-wave or b-wave response in miR-155 KO mice when compared with that in the WT control (Fig. 2h–i). Overall, within the parameters investigated, no changes were observed between the normal and miR-155 KO adult mouse retina.

### MiR-155 KO Results in Mild Protection in the Retina in Response to PD

**Fig. 3:**
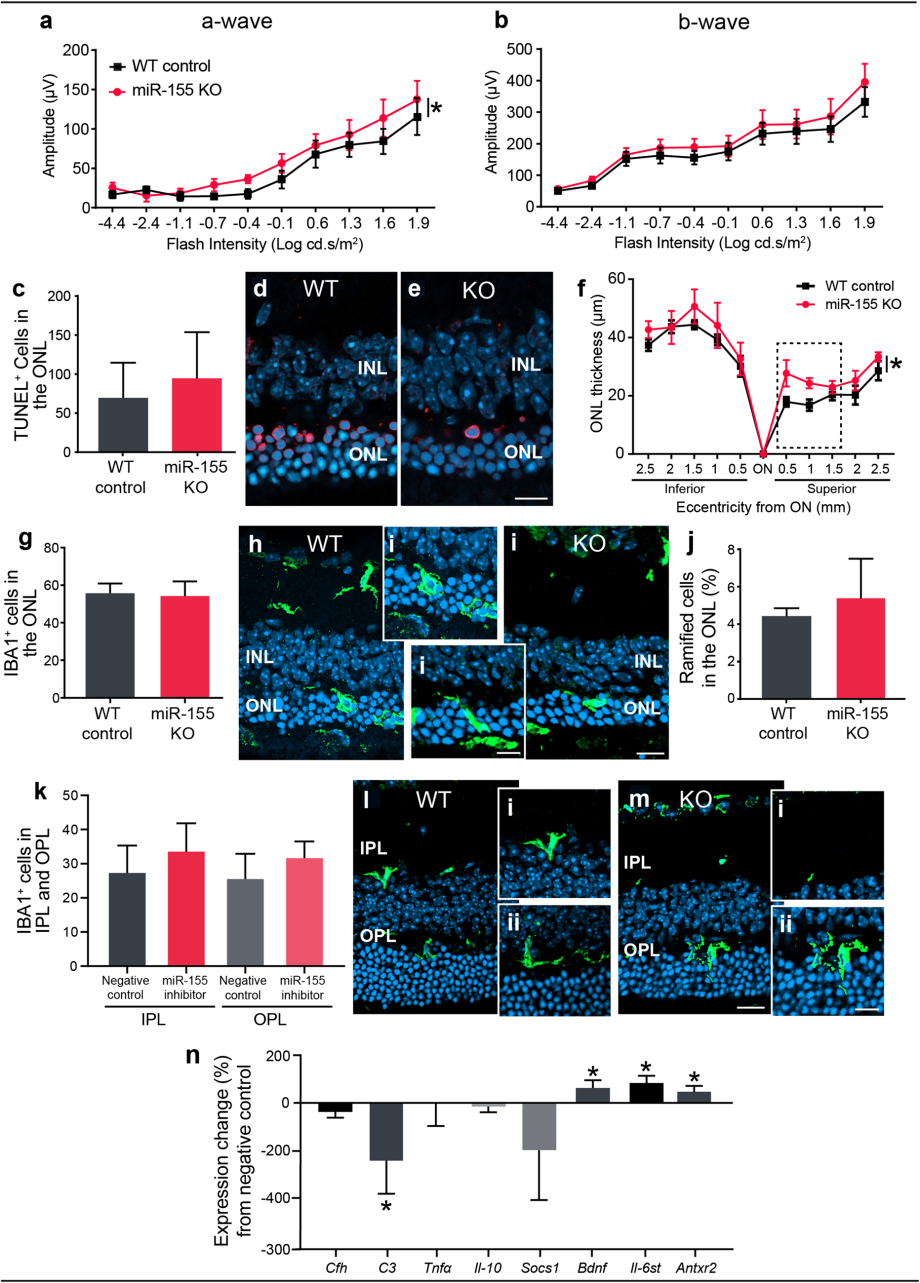
miR-155 KO mouse retinas demonstrated some protection against photo-oxidative damage (PD). **a**–**b** Retinal function of miR-155 KO and WT retinas at 5 days PD (**a**) demonstrated a significantly higher a-wave amplitude in the miR-155 KO (**b**) and no significant difference in b-wave amplitude between the miR-155 KO and control (*p* < 0.05). **c**–**e** Quantification of photoreceptor cell death by TUNEL immunolabelling of retinal cryosections showed no significant difference in TUNEL^+^ cells between groups. **f** ONL thickness across the length of the retina was significantly thicker in miR-155 KO mice compared with that in WT controls (*p* < 0.05). The box indicates the region of focal cell death. **g** IBA1^+^ cells were quantified and compared between miR-155 KO and control mice showing no significant difference in total counts at 5 days PD. **h**–**i** Representative images of IBA1^+^ cells in the ONL. **j**–**m** There was no difference in the percentage of ramified cells in (**j**) the outer retina or (**l**–**m**) plexiform layers between miR-155 KO and WT mice (*p* > 0.05). **n** The gene expression of complement (*Cfh*, *C3*), general cytokines (*Il-10*, *Tnfα* and *Socs1*) and predicted targets of miR-155 (*Bdnf*, *Il6st* and *Antxr2*) was measured by qRT-PCR. There was no change in the expression of *Cfh*, *Tnfα*, *Il-10* and *Socs1*;however, there was a significant decrease in the downstream complement component *C3* (*p* < 0.05). Additionally, *Bdnf*, *Il6st* and *Antxr2* showed a significant increase in expression in miR-155 KOs compared with that in controls (*p* < 0.05). Statistical significance was determined by Student’s *t* test and two-way ANOVA with post hoc multiple comparison (*n* = 10 animals per group, *represents *p* < 0.05). ONL, outer nuclear layer; INL, inner nuclear layer; IPL, inner plexiform layer, OPL; outer plexiform layer. For all images, scale bars represent 20 μm (Hi, Ii: scale bar represents 10 μm)

To verify the observed effects of miR-155 inhibition and to further elucidate the role of miR-155 in the degenerating retina, miR-155 KO mice were subjected to 5 days of photo-oxidative damage, following which functional and histological measures were taken. ERG results showed a significantly larger a-wave response but no statistical difference in b-wave response across increasing flash intensities in miR-155 KO photo-oxidative damage mice compared with that in WT controls (Fig. 3a–b). To explain the functional preservation observed in miR-155 KO mice, photoreceptor cell death was compared between the miR-155 KOs and WT controls by TUNEL assay. However, no difference was found in TUNEL^+^ cell counts between groups (Fig. 3c–e). Cumulative photoreceptor cell death was determined by measuring ONL thickness across the entire retina, showing that the ONL was significantly thinner in the WT controls than in the miR-155 KO (Fig. 3f).

Quantification of IBA1^+^ MPs accumulating in the outer retina (ONL and SRS) and plexiform layers was used as a measure of inflammation. There was no decrease in the number of IBA1^+^ cells in the outer retina (Fig. 3g–i). Furthermore, there was no difference in the morphology of MPs in the outer retina (Fig. 3j). Similarly, MPs in the plexiform layers showed no difference between the controls and miR-155 KOs (Fig. 3k–m). To further investigate retinal inflammation in miR-155 KO retinas, the expression of inflammatory cytokines and potential targets of miR-155 (see Supplementary Table 2) was measured by qRT-PCR. The expression of *Cfh* did not increase in the miR-155 KO compared with that in the WT control following photo-oxidative damage; however, there was a significant, ~ 200% decrease in the expression of *C3* in the KO retinas. While there was no difference in *Tnfα*, *Il-10* or *Socs1* expression, there was a significant increase in the expression of *Bdnf*, *Il-6st* and *Antxr2* (Fig. 3n). In summary, there was some protection of function and photoreceptor survival in the miR-155 KO retinas in comparison with that in the WT controls; however, this did not translate into a no clear difference in counts or morphology of inflammatory cells.

### MiR-155 Is Predominantly Localised to the Nucleus of Retinal Macroglia and Microglia in the Normal Retina

**Fig. 4:**
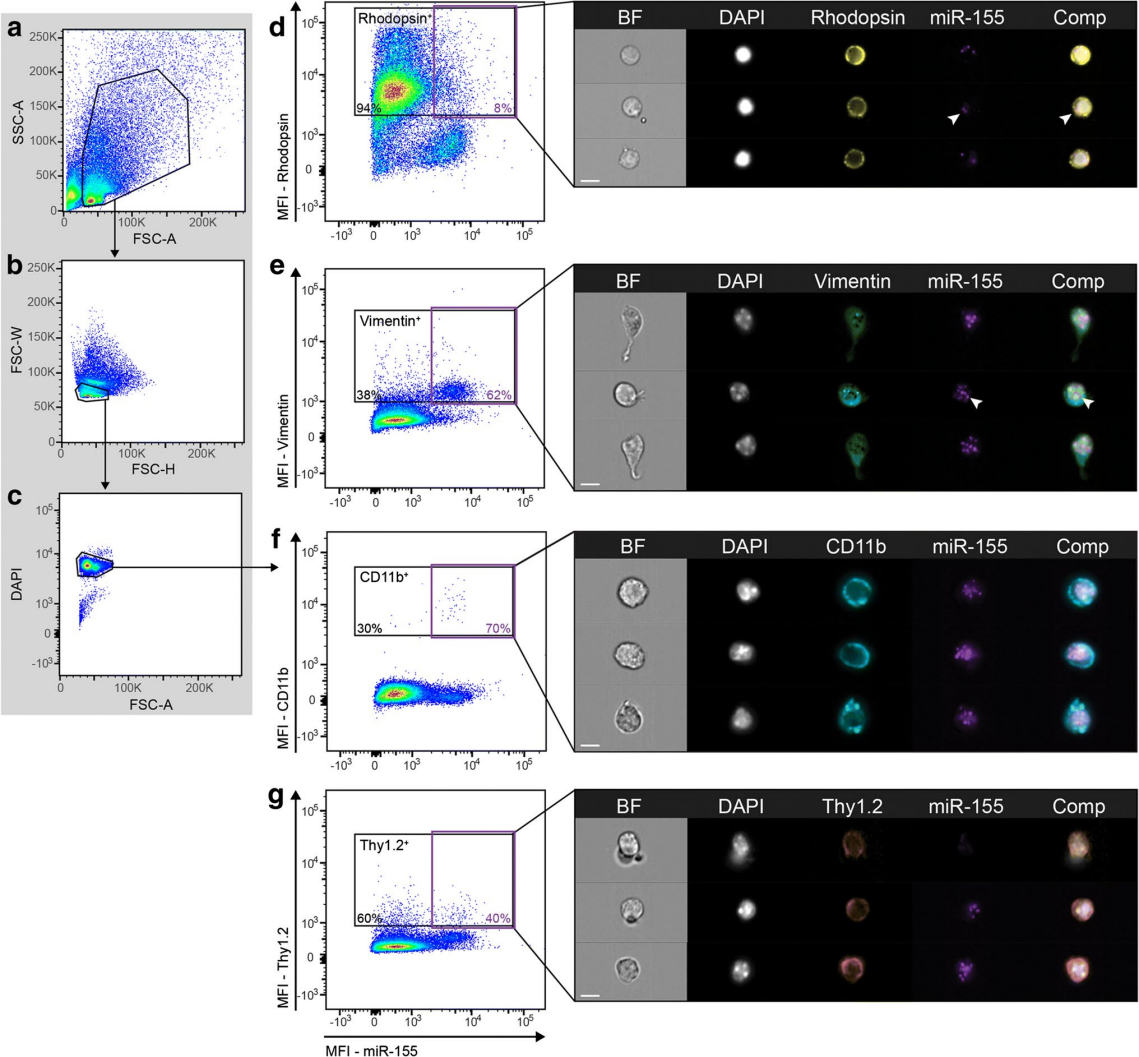
miR-155 expression was predominantly detected in glia of DR WT retinas. **a**–**g** Retinal cell suspensions were stained for lineage markers using fluorophore-conjugated antibodies and miR-155 expression using a PrimeFlow assay. **a**–**c** Single, DAPI-positive retinal cells were gated on by flow cytometry. **d**–**g** Cell populations were identified using lineage markers, rhodopsin (photoreceptors), vimentin (glia), CD11b (microglia) and Thy1.2 (CD90.2; ganglion cells). **d** Flow cytometric analysis revealed approximately 6% of Rhodopsin^+^ cells to be positively stained for miR-155. ImageStream visualisation shows miR-155 staining to be primarily located within the cytoplasm of Rhodopsin-miR-155 double-positive cells (arrow). **e** Approximately 62% of vimentin^+^, CD11b^−^ cells were positive for miR-155. The brightest miR-155 staining occurred in vimentin^+^ cells. **f** Approximately 70% of CD11b^+^ cells were miR-155-positive. **g** Approximately 35% of Thy1.2^+^ cells showed miR-155 expression. miR-155 fluorescence was detected in the nucleus and cytoplasm of miR-155 positive, vimentin^+^, CD11b^+^ and Thy1.2^+^ cells. *n* = 3 samples per group with 4 pooled retinas per sample. For all images, scale bars represent 7 μm

To determine the cell-specific localisation of miR-155 in the normal retina, retinal single-cell suspensions from dim-reared, WT, C57BL/6J mice were stained for miR-155 expression using PrimeFlow™. Cells were then double-labelled with lineage markers of four major retinal cell types: photoreceptors (rhodopsin); Müller glia and astrocytes (vimentin); ganglion cells (thy1.2); and MPs (CD11b). Single, DAPI^+^ cells were gated for by flow cytometry to remove cell aggregates from analysis (Fig. 4a–c). As the isolation of MPs has previously shown contamination with rod photoreceptor–specific transcripts [70, 71], rod photoreceptors were identified based on the expression of rhodopsin in combination with an inverted nuclear pattern of DAPI staining (Fig. 4d). Rhodopsin^+^ photoreceptors exhibited little to no miR-155 fluorescent staining, with only 6% of rhodopsin^+^ cells positively stained for miR-155. Furthermore, miR-155 fluorescence in these cells was rarely localised to the nucleus, but rather, was found to be predominantly localised in the cytoplasm (Fig. 4d). Expression of miR-155 was found in 62% of vimentin^+^ CD11b^−^ cells (Fig. 4e), 70% of CD11b^+^ cells (Fig. 4f) and approximately 40% of thy1.2^+^ ganglion cells (Fig. 4g). Within these cell types, miR-155 expression appeared to largely localise within the nucleus, as indicated by colocalisation with the nuclear stain, DAPI. Overall, these results demonstrate the greatest abundance of miR-155 transcript to be within macroglia (vimentin^+^, CD11b^−^) and microglia (vimentin^+^, CD11b^+^) cells.

### MiR-155 Expression Is Produced by Macroglia and MPs under Stress

**Fig. 5:**
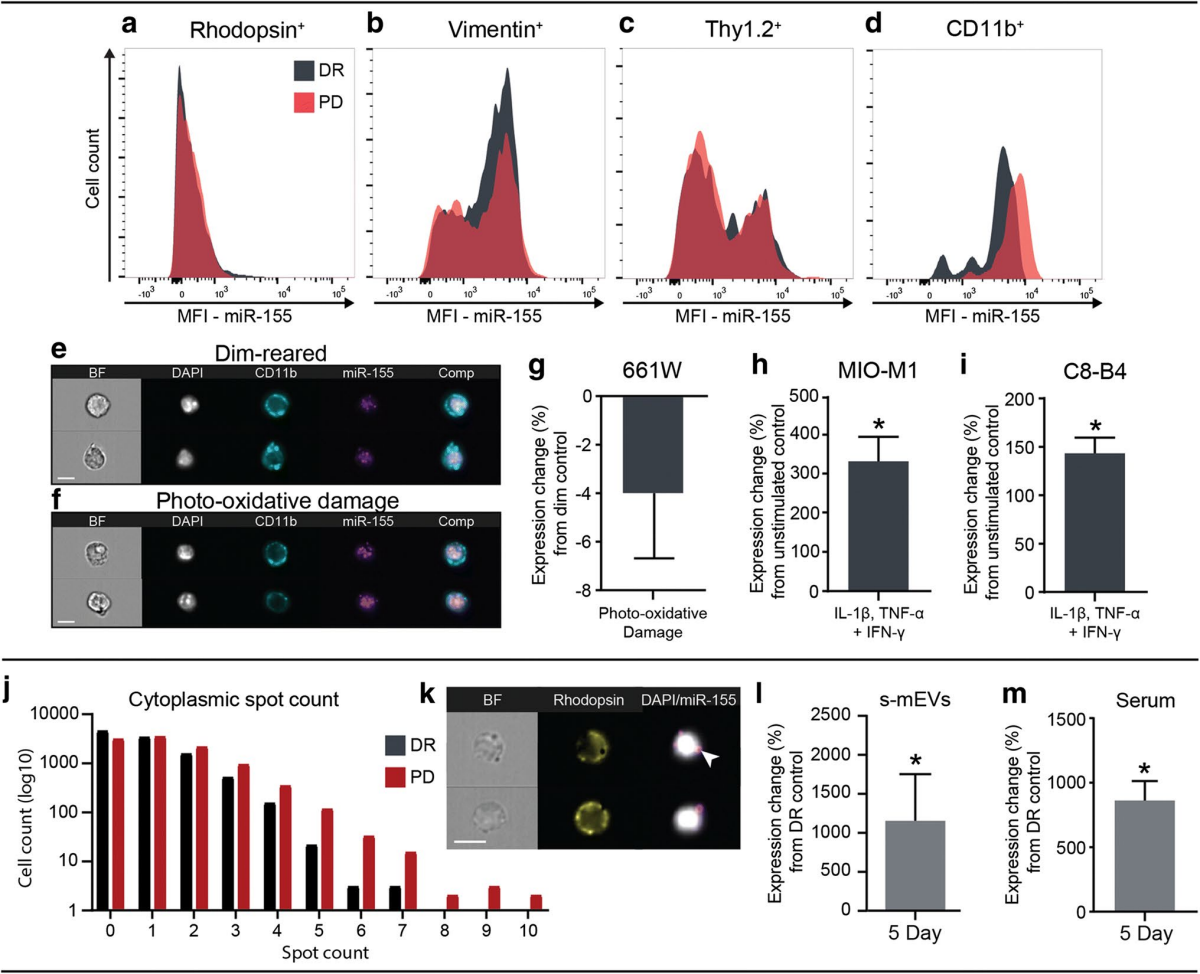
miR-155 in primary and immortalised retinal cells following stimulation. **a**–**d** Flow cytometric analysis of miR-155 expression demonstrated no difference in (**a**) rhodopsin^+^ cells (**b**) vimentin^+^ cells or (**c**) thy1.2^+^ cells before and after PD. (**d**) There was an increase in miR-155 mean fluorescence intensity (MFI) in CD11b^+^ cells following PD. **e**–**f** Representative images of miR-155 labelling by AF-647 in (**e**) DR and (**f**) 5-day PD CD11b^+^ cells. **g**–**i** Immortalised cell lines, 661W (photoreceptors), MIO-M1 (Müller glia) and C8-B4 (brain-derived microglia) were stimulated to mimic a degenerative response. **g** There was no change in miR-155 expression in 661W photoreceptor-like cells following PD compared with dim controls (*p* > 0.05); however, a significantly greater expression of miR-155 was seen in both (**h**) MIO-M1 and (**i**) C8-B4 cultures following stimulation with a cytokine cocktail compared with unstimulated controls (*p* < 0.05). **j** Quantification of miR-155 in rhodopsin^+^ photoreceptors showed more cells with no detectable cytoplasmic miR-155 in DR compared with PD and consequently, more rhodopsin^+^ cells with a higher miR-155 spot count following PD. **k** Representative image indicating miR-155 located in the cytoplasmic space of rhodopsin^+^ photoreceptors. **l** miR-155 expression, measured by qPT-PCR, was significantly higher in s-mEV isolated from PD retinas compared with that in DR controls (*p* < 0.05). **m** MiR-155 expression was also significantly increased in the serum of mice following PD (*p* < 0.05). Statistical significance was determined by Student’s *t* test (*n* = 3–4 samples per group with 4 pooled retinas per sample, *represents *p* < 0.05). For all images, scale bars represent 7 μm

To further elucidate the role of miR-155 in the WT degenerating retina, the expression of miR-155 was assessed in four major retinal cell types by flow cytometry using a PrimeFlow™ assay. We compared 5-day photo-oxidative damage retinas with dim-reared control retinas. There was no change observed in the expression of miR-155 in rhodopsin^+^, vimentin^+^ or thy1.2^+^ cells (Fig. 5a–c). However, there was a slight increase in the fluorescence intensity of miR-155 in the CD11b^+^ cells as a result of photo-oxidative damage (Fig. 5d–f). We aimed to corroborate these findings by exploring miR-155 expression in vitro.

Immortalised cell lines 661W (photoreceptor-like cells), MIOM1 (Müller glia-like cells) and C8B4s (brain-derived microglia) were stimulated with photo-oxidative damage or a cytokine cocktail and miR-155 expression measured by qRT-PCR. There was no change in miR-155 expression in 661W cells following 5-h photo-oxidative damage compared with dim controls (Fig. 5g). However, miR-155 was significantly increased in both MIO-M1 and C8-B4 cells following a 24-h cytokine cocktail challenge (Fig. 5h–i). Therefore, a cytoplasmic mask combined with a spot-counting feature was used for in-depth analysis of the miR-155 cytoplasmic content of rhodopsin^+^ cells in dim-reared and photo-oxidative damage retinas. We found a greater proportion of cells containing miR-155 in the photo-oxidative damage population than the dim-reared, together with photo-oxidative damage rhodopsin^+^ cells displaying a greater number of cytoplasmic spots (Fig. 5j–k). The absence of miR-155 in the nucleus, combined with the cell culture results, indicates that miR-155 is likely not produced by photoreceptors. In summation, this suggests that cytoplasmic miR-155 is translocated to photoreceptors at an increased frequency during photo-oxidative damage.

To assess the potential mechanism by which miR-155 may be communicated to photoreceptors, the level of miR-155 in retinal s-mEVs of WT mice before (dim-reared) and after photo-oxidative damage was assessed. There was a significant increase in miR-155 in s-mEV isolated from 5-day photo-oxidative damage retinas compared with photo-oxidative damage controls (Fig. 5l). Furthermore, we measured miR-155 in systemic serum to add validity to s-mEV loading, demonstrating an increase in miR-155 at 5 days photo-oxidative damage when compared with that in dim-reared controls (Fig. 5m). Taken together it appears that miR-155 is acting as a signalling molecule produced locally by macroglia and MPs, and possibly exported in s-mEV which are delivered to photoreceptors and systemic circulation under degenerative conditions.

### Combinational Treatment with miR-155 and miR-146a Inhibitors Presents No Therapeutic Efficacy Against Retinal Degeneration

It is well-documented that miR-155 and miR-146a act on similar pathways in inflammation with overlapping binding sites on the 3′UTR of multiple mRNA targets, creating some redundancy in regulatory pathways [21, 24, 28]. We therefore wanted to determine the therapeutic potential of modulating two miRNAs in the retina, miR-155 and miR-146a to reduce inflammation and degeneration.

Firstly, the expression of both miR-155 and miR-146a was measured over a time course of 1, 3 and 5 days photo-oxidative damage by qRT-PCR to determine potential peaks in miRNA abundance, compared with a dim-reared control (Supplementary Fig. 2a–b). A significant increase in miR-155 and miR-146a was identified at all three time points. As a known target of both miRNAs, and a key molecule associated with retinal degeneration [72], the regulation of CFH was confirmed using a luciferase assay. A significant, ~ 2-fold reduction in the relative luciferase activity for CFH was observed with the addition of miR-155 or miR-146a (Supplementary Fig. 2c). Furthermore, the combination of miR-155 and mir-146a demonstrated a significantly larger decrease in relative luciferase activity then miR-155 alone, although not miR-146a. Given that the combination demonstrated a reduction in luciferase activity from a negative miRNA inhibitor control and early photo-oxidative damage time points demonstrated marked increase in miR-155 (1 and 3 days) and miR-146a (3 days), we chose to intravitreally administer inhibitors of both miRNAs at day 2 during a 5-day photo-oxidative damage period to assess their combinational therapeutic efficacy. At 5-day photo-oxidative damage, we assessed retinal function by ERG, comparing with retinas injected with a scrambled miRNA control. We observed no change in a-wave, but a significantly lower b-wave response when compared with the scrambled miRNA controls (Supplementary Fig. 2d–e). Neuronal cell death at 5 days photo-oxidative damage was assessed by TUNEL assay demonstrating no difference when treated with miRNA inhibitors (Supplementary Fig. 2f–h). Additionally, there was no change in the cumulative cell death of photoreceptors as indicated by ONL layer thickness between these groups (Supplementary Fig. 2i). However, there was a significant increase in the number of IBA1^+^ cells, specifically in the ONL when compared with scrambled controls (Supplementary Fig. 2j–l). Due to the combined CFH suppression by miR-155 and miR-146a in vitro, *Cfh* and the central complement molecule *C3* were measured by qRT-PCR. There was no significant difference in the expression of *Cfh* or *C3* between the scrambled miRNA inhibitor control and combined inhibition of miR-155 and miR-146a (Supplementary Fig. 2m).

Due to the combination therapy demonstrating no protective effect on the photo-oxidative damage rodent retinas, and to investigate individual roles, we focused our investigations on targeting miR-146a individually.

### Treatment with miR-146a Inhibitor Alone Offered No Protective Effect

To determine the therapeutic potential of miR-146a inhibition, miR-146a inhibitors were intravitreally injected into the retina at 2 days during a 5-day photo-oxidative damage period. Retinal function was assessed by ERG, revealing that there was no difference in function for either a-wave or b-wave when compared with scrambled miRNA inhibitor controls (Supplementary Fig. 3a–b). Further histological analysis demonstrated no change in photoreceptor cell death at 5 days photo-oxidative damage, indicated by TUNEL^+^ cell counts, and cumulative cell death, assessed by ONL thickness (Supplementary Fig. 3c–f). Additionally, no difference was observed between miR-146a inhibitor and scrambled inhibitor control–treated retinas in IBA1^+^ counts of MP infiltration into the outer retina (Supplementary Fig. 3g–i). The expression of complement genes *Cfh* and *C3*, measured by qRT-PCR, remained unchanged in response to miR-146a inhibition (Supplementary Fig. 3j). Taken together, the inhibition of miR-146a did not appear to protect against degeneration in the photo-oxidative damage retina.

## Discussion

Although a role for miR-155 and miR-146a in degenerative diseases of the central nervous system (CNS) has been described [24, 31, 49, 51, 73], their role remains relatively underexplored in the retina. Furthermore, biological targets of miR-155 and miR-146a in retinal degenerations have yet to be fully explored. This study notably demonstrated that inhibition of miR-155 provides retinal protection, particularly in alleviating the typical immune response during retinal degenerations. We further show, for the first time in the retina, the localisation of miR-155 to the nucleus of macroglia and MPs and at low levels in the cytoplasm of photoreceptors. We believe that this facet may be due to the transport of mature miR-155 miRNA strands within the retina, potentially in an s-mEV-dependent manner. Assessment of a miR-155 KO to verify findings revealed no change in inflammation, suggesting retinal protection is occurring by the regulation of additional pathways. This study demonstrated that the inhibition of miR-155 in the retina offers functional protection; however, the specific retinal mRNA targets or molecular interactions remain elusive.

### Inhibition of miR-155 Attenuates MP-Mediated Inflammation in Retinal Degenerations

The inhibition of miR-155 in the CNS has been shown previously to be beneficial against neuroinflammation by alleviating MP reactivity [42, 44–46, 49, 69, 74, 75]. A characteristic homeostatic, or ‘alternatively activated’ morphology has further been demonstrated in MPs as a consequence of miR-155 ablation in a superoxide dismutase (SOD) mouse model of ALS, reversing the abnormal gene expression profile [69]. Similarly, it was demonstrated here that MPs in the ONL of photo-oxidative damage retinas exhibited a more ramified morphology when treated with a miR-155 inhibitor. This appearance is characteristic of ‘alternatively activated’ MPs associated with maintenance and repair of the retina [76]. We therefore consider that although inhibition of miR-155 did not change the number of MPs in the outer retina, the observed retinal protection may be due to a modulation of processes such as chemokine signalling as demonstrated in previous studies in the retina [77–80].

miR-155 was identified to potentially act in a positive-feedback loop with pro-inflammatory cytokines including TNF-α, propagating MP-mediated inflammation and subsequent retinal degeneration [77–79]. Furthermore, in alignment with the work by Jablonski et al. (2016) demonstrating that inhibition of miR-155 reduces MP activation and pro-inflammatory cytokine production [80], we demonstrated a decrease in *Tnf-α* in conjunction with a typically homeostatic MP morphology in response to miR-155 inhibition. We believe that this decrease in pro-inflammatory activation of MPs would also contribute to the reduction of aberrant functions such as phagocytosis.

While phagocytosis is part of the normal transient inflammatory response in the retina, the persistent presence of pro-inflammatory MPs in the outer retina is known to further hamper retinal function by increasing photoreceptor cell death via phagocytosis of stressed, but viable cells [81]. This deleterious process occurs in part via the loss of ‘do not eat me’ signals on photoreceptors, for example CD47, a target of miR-155 [82, 83]. While the direct interaction of miR-155 with these targets has not yet been explored in the retina, we suggest that miR-155 could contribute to ONL thinning in two parts, firstly by increasing MP activation and phagocytic capacity and secondly via the regulation of ‘do not eat me’ photoreceptor signals. Subsequently, we hypothesise that inhibition of miR-155 attenuates this pro-inflammatory or ‘classical’ activation response of MPs in the photo-oxidative damage retina leading to increased photoreceptor survivability. However, although the phenotypic measure used here provides an indication of MP state, supplementary assessment using homeostatic and cell lineage markers of MP populations in the degenerating retina would provide essential validation to the MP response to miR-155 inhibition.

In addition to these phenotypic changes, we observed an increase in the number of MPs within the plexiform layers of miR-155 inhibitor–treated retinas at 5 days photo-oxidative damage. Where MPs in the outer retina are associated with the diseased state, Wang et al. (2016) have shown that steady state microglia located in the plexiform layers are required for the maintenance of synapse structure in the outer retina and synaptic transmission [84]. Furthermore, the ablation of MPs alone has been shown to be sufficient in reducing ERG function over an extended time course of 30 days. We therefore postulate that protection of retinal function observed here in conjunction with significantly more MPs in the plexiform layers may be due to a similar mechanism of preserving the maintenance of synapses. However, further investigations into the mechanism underlying the increased MP population in the plexiform layers are required to fully understand this effect.

### MiR-155 Is Expressed by Glial Cells of the Retina in Response to Stress

Due to low abundance of miR-155 within the retina, the localisation of miR-155 by traditional methods, e.g. in situ hybridisation, has proven to be ineffective [20]. Here, we used a combination of PrimeFlow™ assay, measured by traditional flow cytometry and imaging flow cytometry, and cell culture to gain an understanding of how miR-155 is expressed within the retina. Analysis of miR-155 expression by flow cytometry in mouse isolated photoreceptors, macroglia (Müller cells and astrocytes), MPs and ganglion cells demonstrated an overall increase in miR-155 solely in the CD11b^+^ MP population following photo-oxidative damage. Transcript expression measures of miR-155 in response to stimulation confirmed this upregulation of miR-155 by MPs in addition to a demonstrated miR-155 production by Müller glia, these principal glia cells of the retina, functionally akin to astrocytes of the brain [85]. This expression by macroglia in culture may not have been detected in retinal photo-oxidative damage samples due to miR-155 being released at an earlier time point, the use of a human cell line, or that the pathway required for miR-155 maturation and release is not active in macroglia in the photo-oxidative damage retina. Regardless, taken together, these findings align with previous investigations into miR-155 abundance in the hippocampus, showing miR-155 to increase in regions of both microglia and astrocyte activation following stress [86]. Likewise, primary microglia derived from cortical tissue have shown increased mir-155 expression in response to stress [44]. The upregulation of miR-155 has subsequently been linked to an impedance of astrocyte and microglia function in the brain [46, 49, 69]. Therefore, we believe miR-155 to be similarly involved in the stress response in retinal glia.

### MiR-155 Potentially Translocates Between Glia and Neurons in an s-mEV Dependent Manner

While it was shown that miR-155 is expressed by MPs under stress and contributes to the MP inflammatory response in retinal degenerations, our data suggests that miR-155 is also taken up by rhodopsin^+^ photoreceptors, particularly during degeneration. This is considered due the absence of nuclear miRNA expression, as PrimeFlow is not specific to the mature miRNA strand, nuclear staining likely indicates presence of the pri- or pre-miRNA expressed by the host cell. Similar to the findings presented here, Koval et al. (2013) previously showed that administration of fluorescently tagged anti-miR-155 administration to the lateral ventricle of the brain induced cell specific uptake by neurons, microglia and astrocytes, leading to the regulation of miR-155-directed activity [87]. Here, it is hypothesised that miR-155 can influence markers expressed by photoreceptors to encourage loss of function and survivability, such as ‘do not eat me’ signals; however, the mechanism by which this transport occurs is currently unknown.

Intercellular communication of miRNA is essential for normal tissue functioning, and interruptions of this process are shown to induce neuronal deficits [88]. Previous studies have identified the inhibition of glia-neuron directional communication of miR-155 in a culture system to increase neuronal viability [44]. Furthermore, the signalling of exosome-encapsulated pro-inflammatory miRNA from glia to neurons can be detrimental to neuronal synapses [89]. Here, we identified increased miR-155 expression within isolated retinal s-mEVs, as a consequence of photo-oxidative damage. Additionally, miR-155 fluorescence was detected to increase in the cytoplasm of rhodopsin^+^ photoreceptors, which were virtually devoid of nuclear staining for miR-155, indicating an absence of pri-miRNA. Therefore, we consider the miR-155 detected in photoreceptors to be derived from miR-155 expressing cells, such as macroglia and MPs and subsequently transported to photoreceptors in an s-mEV-dependent manner. Moreover, we hypothesise that the increased detection of miR-155 in circulation, aligning to previous findings [31], is due to the activation of circulating monocytes and/or the capability of s-mEVs to cross blood-CNS-barriers [90]. These features of s-mEV-directed communication of miR-155 have led to its investigation as both diagnostic and therapeutic [91].

Taken together, we speculate that the combination of retaining plexiform layer MPs and the reduction in miR-155 signalling protects against neuronal cell death and maintains synapse integrity. However, investigation into synapse structure and function as well as further exploration into cell-to-cell communication in the photo-oxidative damage retina is required for a more in-depth understanding of this process.

### Depletion of miR-155 Demonstrates Potential Redundancy in Inflammatory Pathways Suggesting Other Mechanisms of Protection

It was relatively unexpected that the protective effect of miR-155 inhibition did not fully translate to miR-155 KO rodent retinas. Although previous studies have identified slight alterations to retinal vasculature in the miR-155 KO mouse [74], we identified no significant histological or functional deficiencies in the dim-reared retina. Additionally, although there was an observed protection of function and photoreceptor viability, in the photo-oxidative damage retina, we did not identify a change in inflammation. This is contrary to previous studies that have identified a greater occurrence of homeostatic microglia in miR-155 KO retinas following oxygen-induced retinopathy [74]. Similarly, Woodbury et al. (2015) documented decreased microglia activation in the brains of miR-155 KO mice in response to lipopolysaccharide (LPS)-induced inflammation [92]. Escobar et al. (2013) also showed a reduction of inflammatory cells in the vitreous and retina of miR-155 KO mice with experimental autoimmune uveitis (EAU) [93]. Granted, the discrepancy between findings could be due to subtleties in disease pathology, however, we postulate that compensatory mechanisms may also be influencing the inflammatory potential in the KOs. The knockdown of a single miRNA can result in the upregulation in other miRNA family members with overlapping binding sites on the target mRNA. Over a lifespan, these pathways have time to adjust creating redundancy within clusters [94]. Although we see reduction in potential AGO2-mediated binding mRNA transcripts, there is no change in indicators of broader inflammatory pathways and the location and phenotype of immune cells in the KO retina. We therefore suggest a greater redundancy in inflammatory pathways with regard to miR-155 and indicate the regulation of an alternative protective mechanism for neuronal survival and functional protection.

Investigations by Harrison et al. (2017) in the rodent hippocampus in an acute model of cortical impact, identified localisation of miR-155, via in situ hybridisation, to be largely within neuronal nuclei [95]. While the findings presented here do not show miR-155 expression in the nucleus of the predominant neuronal population, rhodopsin^+^ photoreceptors, nuclear miR-155 was detected in a proportion of retinal ganglion cells (RGCs), neurons responsible for conveyance of visual information to the visual cortex [96]. This study was limited by only investigating the two neuronal subtypes of the retina and this could be a point for further investigation. However, for the purpose of this study, the RGCs became a point of interest due the potential regulation of neurotrophic factors such as BDNF, essential to the survival of RGCs in the retina [97, 98]. While miRNA profiling of apoptosing RGCs has not previously shown differential regulation of miR-155 [99], other studies have identified BDNF as a binding partner of miR-155 [100, 101]. Similarly, we show here by AGO-HITS-CLIP that both miR-155 and *Bdnf* are incorporated into AGO2 in the degenerating retina. Furthermore, our previous findings have demonstrated an increase in AGO2 abundance to occur in the ganglion cell layer (GCL) as a consequence of photo-oxidative damage, suggestive that AGO2-mediated miRNA inhibition is also increasing in the GCL during degeneration [59]. Additionally, a greater abundance of *Bdnf* was identified in the miR-155 KO photo-oxidative damage retina when compared with WT; however, this was not apparent in the miR-155 inhibitor retina. This difference warrants further investigation into the regulation of *Bdnf* across the time course of photo-oxidative damage and measures of neuronal integrity.

### Combinational Inhibition of miR-155 and miR-146a Demonstrates Negative Therapeutic Prospect

Lastly, due to the redundancy of roles for miRNAs in biological systems, combinatorial miRNA therapies have been suggested as an effective strategy for alleviating disease progression [102–104]. Our previous research and findings presented here have shown both miR-155 and miR-146a to be significantly upregulated in response to photo-oxidative-induced damage [32]. However, we found that the combinational inhibition of miR-155 and miR-146a amplifies disease severity, decreasing function and increasing MP recruitment to the outer retina. We suggest that this is due to a disparate role of miR-155 and miR-146a in the regulation of the MP activation state. While both have the capacity to negatively regulate CFH, miR-146a is thought to also act as a negative regulator of inflammation, through suppression of inflammatory activators, such as nuclear factor κB (NF-κB). This negative feedback can contribute to a decrease in the expression of downstream pro-inflammatory factors, including miR-155, and consequently MP activation and retinal degeneration [21, 105]. With this considered, it remains unclear as to why the miR-146a inhibitor retinas did not display a more severe pathology but highlights the complexity of combinational miRNA therapies without knowledge of all the mRNA targets.

### Conclusion

Although the precise mechanisms underlying miRNA function in the retina remain to be disentangled for effective application of combinatorial therapies, we show here, for the first time, the potential for the use of miR-155 in slowing the progression of retinal degenerations. The potential s-mEV pathway of miR-155 from glial cells to photoreceptors identified here presents a platform for more targeted strategies in alleviating the MP-mediated inflammatory response propagated into later stages of disease. Subsequently, in alignment with previous studies, we propose a role for miR-155 in the inflammatory response in degenerative conditions of the CNS.

## Electronic Supplementary Material

ESM 1 (DOCX 15 kb)

ESM 2 (DOCX 16 kb)

ESM 3 (DOCX 409 kb)

ESM 4 (DOCX 955 kb)

ESM 5 (DOCX 614 kb)

## Data Availability

All data generated or analysed during this study is included in the published article, and the datasets found in online repositories. The names of the repository/repositories and accession number(s) can be found in the “Methods” section.

## Abbreviations

AGO: Argonaute
ALS: Amyotrophic lateral sclerosis
AMD: Age-related macular degeneration
ANOVA: Analysis of variance
ANTXR2: Anthrax toxin receptor 2
ANU: Australian National University
ARVO: Association for Research in Vision and Ophthalmology
BDNF: Brain-derived neurotrophic factor
BSA: Bovine Serum Albumin
CD11b: Cluster of differentiation molecule 11b
CD47: Cluster of differentiation molecule 47
cDNA: complementary DNA
CFH: Complement factor H
CNS: Central nervous system
Ct: Cycle threshold
DMEM: Dulbecco’s Modified Eagle Media
DNA: Deoxyribonucleic acid
DR: Dim-reared
EAU: Experimental autoimmune uveitis
ERG: Electroretinogram
FBS: Fetal bovine serum
GAPDH: Glyceraldehyde-3-phosphate dehydrogenase
GCL: Ganglion cell layer
HBSS: Hank’s balanced salt solution
HITS-CLIP: High-throughput sequencing of RNA isolated by cross-linking immunoprecipitation
H&E: Haematoxylin and Eosin
IBA1: Ionised calcium-binding adapter molecule 1
IFN-γ: Interferon γ
IL-10: Interleukin 10
IL-1β: Interleukin 1β
IL-6st: Interleukin 6 signal transducer
IPL: Inner plexiform layer
KO: Knockout
lEV: Large extracellular vesicles
LPS: Lipopolysaccharide
miRNA: microRNA
MP: Mononuclear phagocytes
mRNA: messenger RNA
NF-κB: Nuclear factor κB
ONL: Outer nuclear layer
OPL: Outer plexiform layer
PBS: Phosphate-buffered saline
PD: Photo-oxidative damage
Pre-miRNA: Premature miRNA
Pri-miRNA: Primary miRNA
qRT-PCR: Quantitative real-time polymerase chain reaction
RGC: Retinal ganglion cells
RISC: RNA-induced silencing complex
RPE: Retinal pigment epithelium
RT: Room temperature
SDHA: Succinate dehydrogenase complex subunit A
s-mEV: small to medium extracellular vesicles
snoRNA: small nucleolar RNA
snRNA: small nuclear RNA
SOCS1: Suppressor of cytokine signalling
SOD: Superoxide dismutase
TLR: Toll-like receptor
TNF-α: Tumour necrosis factor α
TUNEL: Terminal deoxynucleotidyl transferase dUTP nick end labelling
UTR: Untranslated region
WT: Wild-type
ΔΔCt: Comparative cycle threshold
