# Correction: Inhibition of microRNA-155 Protects Retinal Function Through Attenuation of Inflammation in Retinal Degeneration

**DOI:** 10.1007/s12035-024-04452-6

**Published:** 2024-09-20

**Authors:** Riemke Aggio-Bruce, Joshua A. Chu-Tan, Yvette Wooff, Adrian V. Cioanca, Ulrike Schumann, Riccardo Natoli

**Affiliations:** 1 The John Curtin School of Medical Research, The Australian National University, Australian Capital Territory, Garran Road, Acton, 2601 Australia; 2 The Australian National University Medical School, Mills Road, Australian Capital Territory, Acton, 2601 Australia

**Correction: Molecular Neurobiology (2020) 58:835-854**

10.1007/s12035-020-02158-z

The original version of this article unfortunately contained an error, specifically in Fig. 1, 3 and Supplementary Fig. 2.

The authors recently noticed that some incorrect representative images (Fig. 1d and 1e) appeared in the published article. These errors occurred due to incorrect image files being linked to the Figure files during manuscript finalisation following the review process and transfer from Molecular Neurodegeneration.

The representative images were correct in our original submission (Fig. 3d and 3e in the original submission) to Molecular Neurodegeneration (MOND-D-20–00326). Therefore, here is the corrected Fig. 1, which contains the representative images from our original submission, Fig. 3d and 3e.

Corrected Figure 1
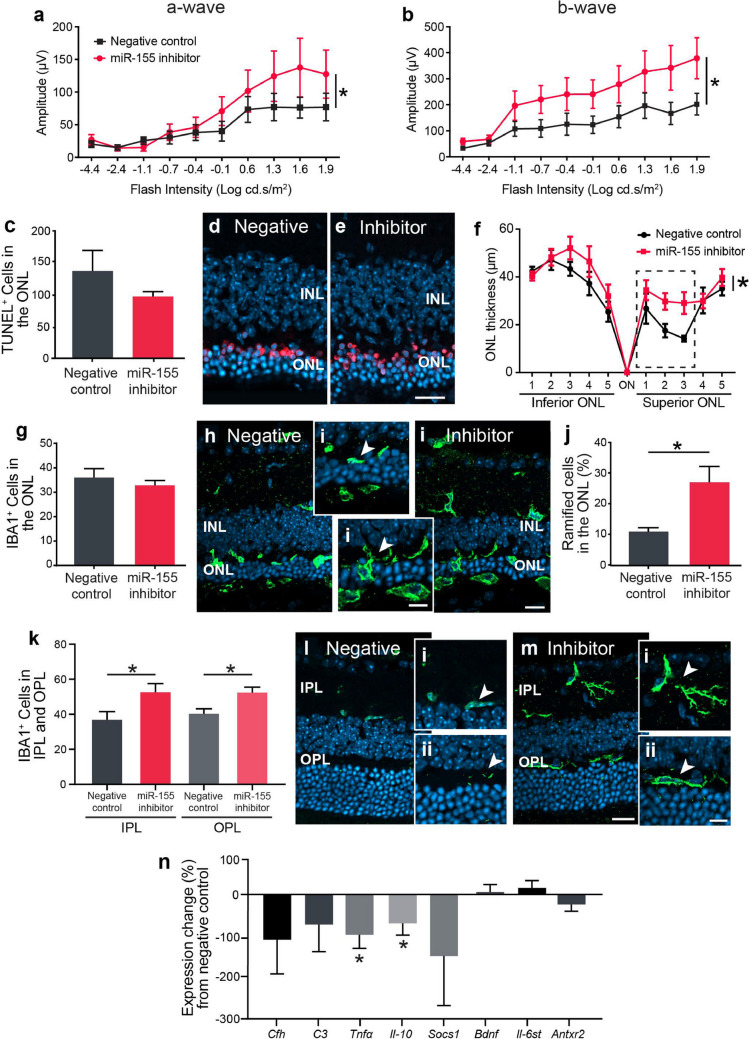


Further, the authors noticed that the same histological image was used as a representative image for two distinct conditions, Fig. 3e and Supplementary Fig. 2h. We have revisited our raw data to identify an alternative representative image for each panel. Below are the corrected Fig. 3 and Supplementary Fig. 2.

Corrected Figure 3
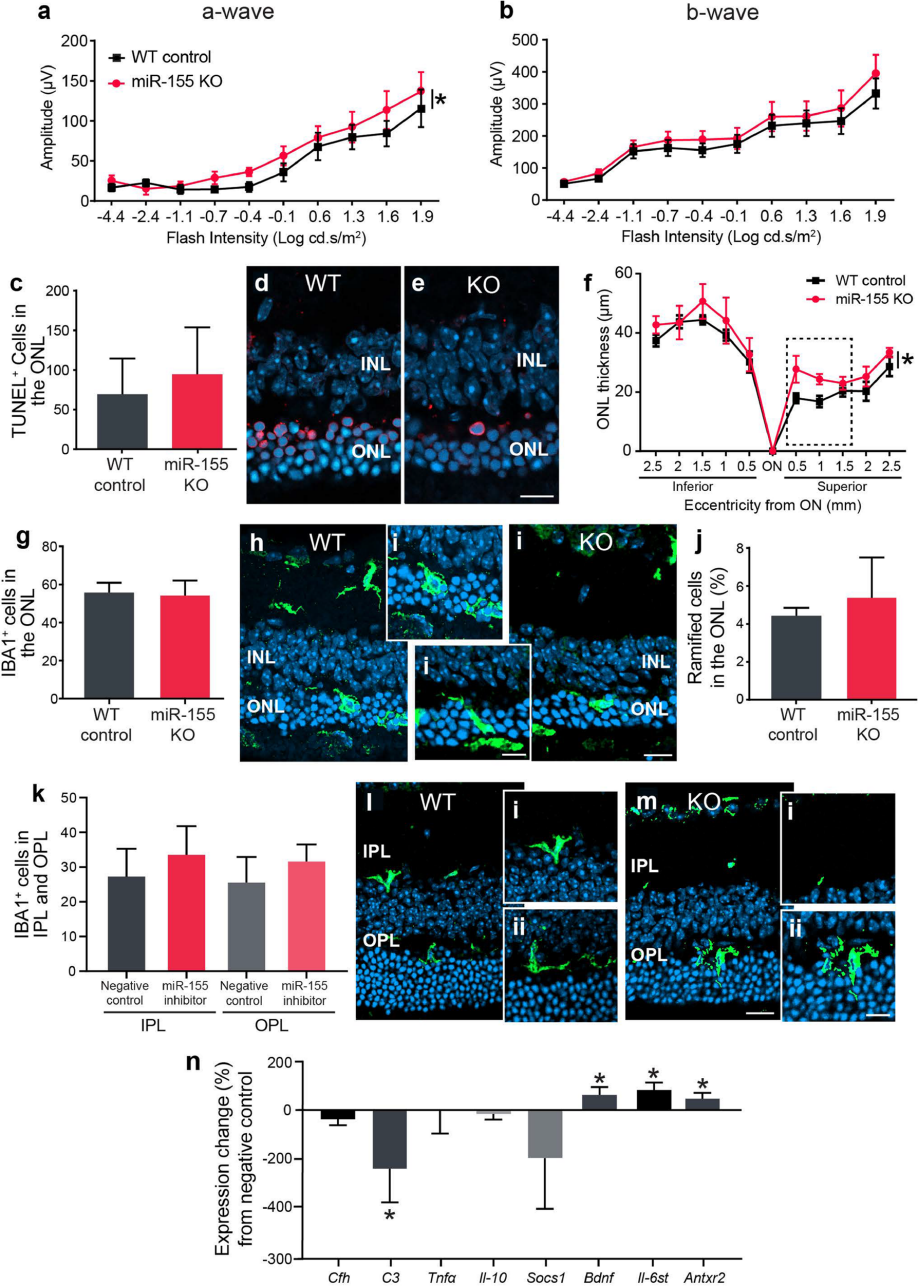


The authors do not consider these changes to alter the findings of the study, however the old images are not an accurate representation of the data, thus must be replaced.

The original article has been corrected.

## Supplementary Information

Below is the link to the electronic supplementary material.

Supplementary file1 (DOCX 956 KB)

