# Supplementary material for: Inhibition of microRNA-155 Protects Retinal Function Through Attenuation of Inflammation in Retinal Degeneration
Source: Mol Neurobiol. 2020 Oct 9;58(2):835–54. doi: 10.1007/s12035-020-02158-z (PMC7843561; doi:10.1007/s12035-020-02158-z)
Supplement: Supplementary file 1 — (DOCX 15 kb) [file 12035_2020_2158_MOESM1_ESM.docx]

**Supplementary Table 1: Targets of miR-155 bound to AGO2 and their differential regulation in photo-oxidative damage.**

| EntrezID | miR-155 targets | logFC | P.Value |
| --- | --- | --- | --- |
| 71914 | **Antxr2** | 2.362633 | 9.70E-09 |
| 17909 | **Myo10** | 1.798979 | 1.95E-08 |
| 16195 | **Il6st** | 1.181876 | 2.54E-06 |
| 13361 | **Dhfr** | -1.16422 | 0.002866 |
| 269881 | **Map3k10** | -2.32351 | 0.008731 |
| ­­­­16485 | **Kcna1** | 0.38997 | 0.01077 |
| 497097 | **Xkr4** | 0.638059 | 0.013658 |
| 12064 | **Bdnf** | -2.16182 | 0.015375 |
| 333433 | **Gpd1l** | 0.548856 | 0.020351 |
| 108934 | **Smim13** | -0.46708 | 0.021471 |
| 108797 | **Mex3b** | -0.69241 | 0.024019 |
| 67263 | **Zswim6** | -0.37465 | 0.025296 |
| 18706 | **Pik3ca** | -1.80816 | 0.026708 |
| 72198 | **Skiv2l2** | -0.67217 | 0.027286 |
| 210710 | **Gab3** | -1.79474 | 0.027589 |
| 114715 | **Spred1** | 0.401608 | 0.030348 |
| 78943 | **Ern1** | 0.466149 | 0.03171 |
| 14284 | **Fosl2** | 0.970243 | 0.032504 |
| 622645 | **Tmem200c** | -0.55575 | 0.036467 |
| 17389 | **Mmp16** | 0.973148 | 0.039864 |
| 12539 | **Cdc37** | 1.726216 | 0.044322 |
| 20254 | **Scg2** | -1.92541 | 0.046604 |
