# Supplementary material for: Inhibition of microRNA-155 Protects Retinal Function Through Attenuation of Inflammation in Retinal Degeneration
Source: Mol Neurobiol. 2020 Oct 9;58(2):835–54. doi: 10.1007/s12035-020-02158-z (PMC7843561; doi:10.1007/s12035-020-02158-z)
Supplement: Supplementary file 2 — (DOCX 16 kb) [file 12035_2020_2158_MOESM2_ESM.docx]

**Supplementary Table 2: Summary of average Ct values of the key investigated targets (rounded to the nearest integer).**

| miRNA | | | | |
| --- | --- | --- | --- | --- |
| *Tissue* | *miR-155* | | *U6* | |
|  | **Dim-reared** | **Photo-oxidative damage** | **Dim-reared** | **Photo-oxidative damage** |
| Retina | 26 | 24 | 17 | 17 |
| Serum | 25 | 23 | 15 | 15 |
| s-mEV’s | 36 | 31 | 25 | 25 |
| 661W | 24 | 25 | 14 | 14 |
| ­­­­MIO-M1 | 27 | 25 | 16 | 16 |
| C8-B4 | 29 | 27 | 18 | 18 |
| ARPE19 | 31 | 30 | 17 | 17 |
| mRNA | | | | |
| *Target* | *Inhibitor* | | *miRNA KO* | |
|  | **Negative inhibitor** | **miR-155 inhibitor** | **Wild-type** | **miR-155 knock-out** |
| *Gapdh* | 22 | 22 | 22 | 22 |
| *Sdha* | 27 | 27 | 27 | 26 |
| *Cfh* | 30 | 33 | 31 | 31 |
| *C3* | 28 | 28 | 27 | 30 |
| *Tnfα* | 27 | 29 | 26 | 27 |
| *Il-10* | 28 | 29 | 26 | 26 |
| *Socs1* | 30 | 30 | 28 | 30 |
| *Bdnf* | 31 | 30 | 29 | 28 |
| *Il6st* | 32 | 31 | 32 | 30 |
| *Antxr2* | 33 | 32 | 32 | 31 |
