# Supplementary material for: Inhibition of microRNA-155 Protects Retinal Function Through Attenuation of Inflammation in Retinal Degeneration
Source: Mol Neurobiol. 2020 Oct 9;58(2):835–54. doi: 10.1007/s12035-020-02158-z (PMC7843561; doi:10.1007/s12035-020-02158-z)
Supplement: Supplementary file 3 — (DOCX 409 kb) [file 12035_2020_2158_MOESM3_ESM.docx]

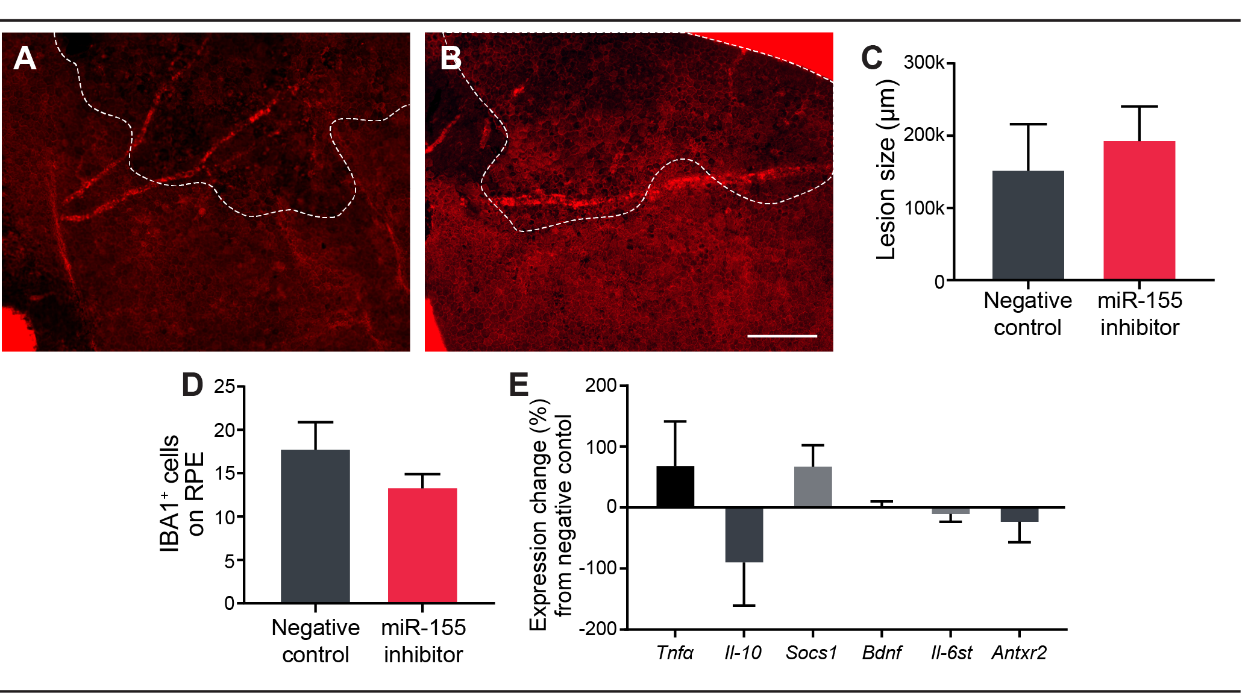


**Supplementary Fig. 1. RPE demonstrated no response to miR-155 inhibition compared to a negative control.**

**a-e** The RPE was compared between eyes intravitreally injected with miR-155 inhibitors or negative controls. RPE whole mounts were stained with phalloidin and IBA-1. **a-b** Representative images showing a portion of the superior lesion on RPE whole mounts. **(c)** Histogram shows no difference in the area of the lesion of miR-155 treated RPE samples compared to a negative control (p > 0.05). **(d)** IBA-1^+^ cells in the sub-retinal space of retinal sections were quantified. The histogram shows no significant difference in IBA-1 positive cell counts (p > 0.05). (**e)** mRNA of miR-155 inhibitor and negative injected retinas were extracted following 5 days PD and analysed by qRT-PCR for changes in the expression of general cytokines (*Il-10, Tnfα* and *Socs1*), and predicted targets of miR-155 (*Bdnf*, *Il6st* and *Antxr2*). No significant differences were measured in miR-155 inhibitor treated RPE compared to the control (p > 0.05). RPE – retinal pigment epithelium. Statistical significance was determined by student t-test (*n* = 10 animals per group, *represents *p* < 0.05). For all images, scale bars represent 200 μm.
