# Supplementary material for: Inhibition of microRNA-155 Protects Retinal Function Through Attenuation of Inflammation in Retinal Degeneration
Source: Mol Neurobiol. 2020 Oct 9;58(2):835–54. doi: 10.1007/s12035-020-02158-z (PMC7843561; doi:10.1007/s12035-020-02158-z)
Supplement: Supplementary file 4 — (DOCX 955 kb) [file 12035_2020_2158_MOESM4_ESM.docx]

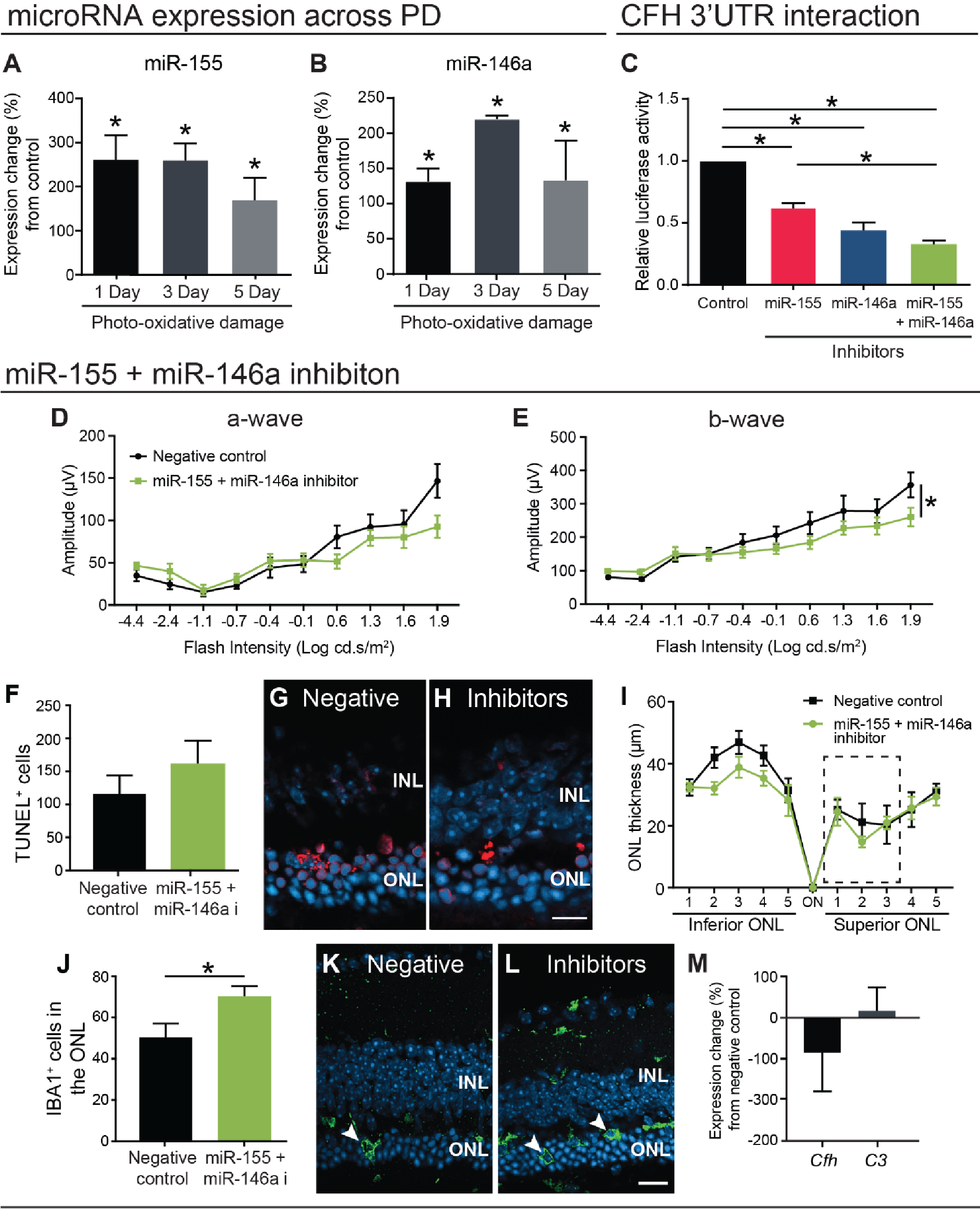


**Supplementary Fig. 2. Inhibition of miR-155 and miR-146a in photo-oxidative damaged (PD) retinas exacerbated degeneration.**

**a-b** Gene expression analysis of miR155 and miR146a across PD using qRT-PCR. Compared to dim-reared (DR) controls both (a) miR-155 and (b) miR-146a were significantly increased across all time points (p < 0.05). **c** A luciferase assay was used in HeLa cells to measure the interaction of miR-155 and miR-146a with the 3’UTR of CFH. CFH luciferase fluorescence was significantly decreased by the addition of each miRNA mimic individually, and was further reduced from miR-155 alone when used in combination (p < 0.05). **d-e** Retinal function as measured by ERG in mice intravitreally injected with miR-155+miR-146a inhibitors compared to negative, scrambled miRNA controls. Mice injected with miR-155+miR-146a inhibitors showed no change in **(d)** a-wave responses, but **(e)** significantly lower b-wave amplitudes compared to controls (p < 0.05). **f** No difference was shown in the levels of photoreceptor cell death as quantified using TUNEL immunolabelling, between treated and control groups (p > 0.05). Representative images indicating TUNEL^+^ cells in the focal lesion site of **(g)** miR-155+miR-146a inhibited and **(h)** negative control mice. (**i)** ONL thickness measured across the length of the retina showed no significant difference between groups (p > 0.05). The box indicates the region of focal cell death. (**j)** IBA1^+^ cells in the ONL were quantified, demonstrating more cells in miR-155+miR-146a inhibited retinas compared to negative controls (p < 0.05). Representative images of ONL IBA1^+^ cells in **(k)** miR-155+miR-146a inhibitor and **(l)** negative control retinas. **(m)** The retinal expression of *Cfh* and *C3,* measured by qRT-PCR was not significantly different from controls following miR-155/miR-146a inhibition (p > 0.05). Statistical significance was determined by student t-test and two-way ANOVA with post-hoc multiple comparison (*n* = 5-10 animals per group, *represents *p* < 0.05). ONL, outer nuclear layer; INL, inner nuclear layer. For all images, scale bars represent 20 μm.
