# Supplementary material for: Inhibition of microRNA-155 Protects Retinal Function Through Attenuation of Inflammation in Retinal Degeneration
Source: Mol Neurobiol. 2020 Oct 9;58(2):835–54. doi: 10.1007/s12035-020-02158-z (PMC7843561; doi:10.1007/s12035-020-02158-z)
Supplement: Supplementary file 5 — (DOCX 614 kb) [file 12035_2020_2158_MOESM5_ESM.docx]

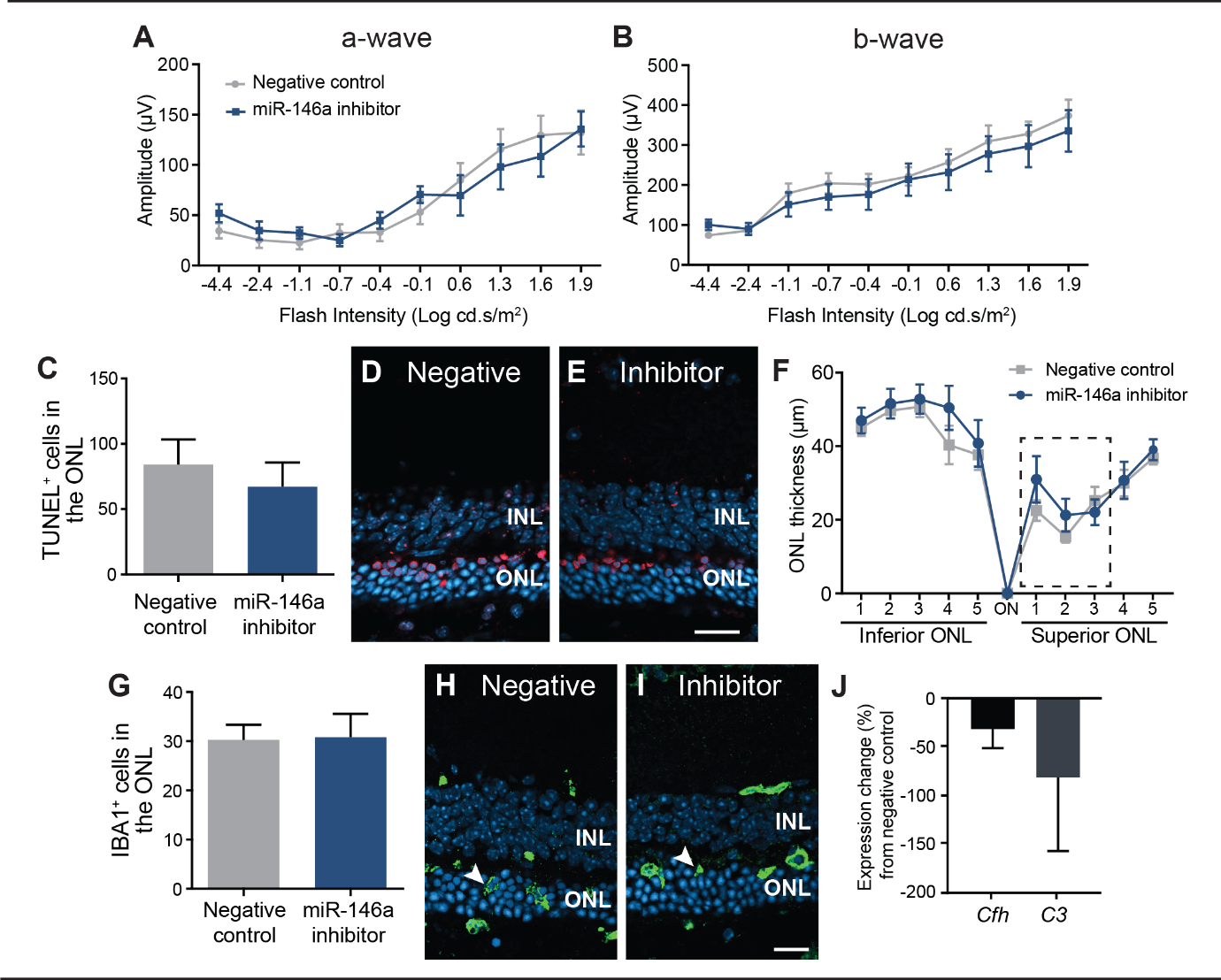


**Supplementary Fig. 3. Inhibition of miR-146a demonstrated no protection against retinal degeneration.** ­

**a-b** Retinal function measured by ERG of miR-146a inhibitor injected compared to negative controls. No significant difference was measured between groups for **(a)** a-wave or **(b)** b-wave measures (p > 0.05). **c-e** Quantification of photoreceptor cell death was performed by TUNEL immuno-labelling of retinal cryosections, showing **(c)** no significant difference in TUNEL^+^ cell counts between miR-146a inhibitor and the control (p > 0.05). **d-e** Representative images displaying no difference in TUNEL^+^ counts. **F** ONL thickness was measured across the retina and showed no significant difference in thickness between miR-146-inhibited mice and controls (p > 0.05). The box indicates the region of focal cell death. **g-i** IBA1^+^ immuno-labelled cells were quantified and compared between miR-146a inhibitor and negative control injected mice. **g** Quantitative analysis of IBA1^+^ cells in the ONL showed no significant difference at 5 days PD (p > 0.05). **h-i** Representative images of IBA1^+^ cells infiltrating the ONL in both miR-146a inhibitor and negative control retinas following PD. **j** There was no significant difference in the retinal gene expression of either *Cfh* or *C3*, as determined by qRT-PCR, following miR-146a inhibition (p > 0.05). Statistical significance was determined by student t-test and two-way ANOVA with post-hoc multiple comparison (*n* = 5-10 animals per group, *represents *p* < 0.05). ONL, outer nuclear layer; INL, inner nuclear layer. For all images, scale bars represent 20 μm.
